# Phosphoinositide-3-Kinase γ Is Not a Predominant Regulator of ATP-Dependent Directed Microglial Process Motility or Experience-Dependent Ocular Dominance Plasticity

**DOI:** 10.1523/ENEURO.0311-20.2020

**Published:** 2020-12-21

**Authors:** Brendan S. Whitelaw, Evelyn K. Matei, Ania K. Majewska

**Affiliations:** 1 Department of Neuroscience; 2 Medical Scientist Training Program and Neuroscience Graduate Program; 3 Center for Visual Science, University of Rochester Medical Center, Rochester, NY 14642

**Keywords:** ATP, chemotaxis, microglia, P2Y12, PI3K, PI3Kγ

## Abstract

Microglia are dynamic cells whose extensive interactions with neurons and glia during development allow them to regulate neuronal development and function. The microglial P2Y12 receptor is crucial for microglial responsiveness to extracellular ATP and mediates numerous microglial functions, including ATP-dependent directional motility, microglia-neuron interactions, and experience-dependent synaptic plasticity. However, little is known about the downstream signaling effectors that mediate these diverse actions of P2Y12. Phosphoinositide-3-kinase γ (PI3Kγ), a lipid kinase activated downstream of G_i_-protein-coupled receptors such as P2Y12, could translate localized extracellular ATP signals into directed microglial action and serve as a broad effector of P2Y12-dependent signaling. Here, we used pharmacological and genetic methods to manipulate P2Y12 and PI3Kγ signaling to determine whether inhibiting PI3Kγ phenocopied the loss of P2Y12 signaling in mouse microglia. While pan-inhibition of all PI3K activity substantially affected P2Y12-dependent microglial responses, our results suggest that PI3Kγ specifically is only a minor part of the P2Y12 signaling pathway. PI3Kγ was not required to maintain homeostatic microglial morphology or their dynamic surveillance *in vivo*. Further, PI3Kγ was not strictly required for P2Y12-dependent microglial responses *ex vivo* or *in vivo*, although we did observe subtle deficits in the recruitment of microglial process toward sources of ATP. Finally, PI3Kγ was not required for ocular dominance plasticity, a P2Y12-dependent form of experience-dependent synaptic plasticity that occurs in the developing visual cortex. Overall, our results demonstrate that PI3Kγ is not the major mediator of P2Y12 function in microglia, but may have a role in amplifying or fine-tuning the chemotactic response.

## Visual Abstract

**Figure:**
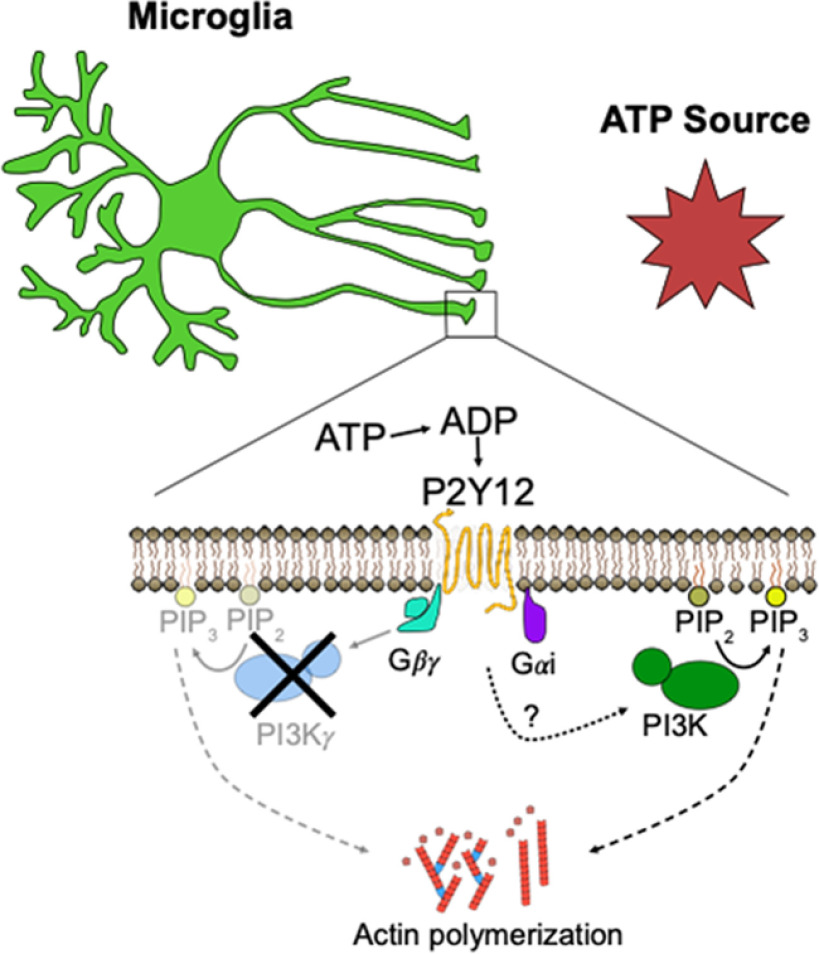

## Significance Statement

Extracellular ATP acts as a chemoattractant for microglial processes via the microglial P2Y12 receptor, facilitating interactions between neurons and microglia. However, the intracellular pathways underlying this directional motility remain unknown. While phosphoinositide-3-kinase (PI3K) activity is required for ATP-mediated microglial motility, PI3Kγ specifically is largely dispensable but plays a subtle modulatory role. Because PI3Kγ is the only isoform directly activated by G-protein-coupled receptors, P2Y12 likely activates PI3K indirectly to promote directed microglial motility.

## Introduction

Microglia, the resident immune cells of the CNS, are critical mediators of neuronal development and function in the healthy brain. Active throughout the lifespan, microglia promote the generation, organization, and connectivity of neurons, and aberrant microglial activity likely contributes to both neuropsychiatric and neurodegenerative diseases (Li and Barres, 2018). Thus, investigation of how microglia interact with their environment is necessary to fully understand neuronal development and disease.

Signaling through extracellular nucleotides has emerged as one of several mechanisms by which microglia communicate and interact with other CNS cells (Vainchtein and Molofsky, 2020). ATP and its hydrolyzis products exhibit profound effects on microglia within minutes (Honda et al., 2001; Davalos et al., 2005), with ADP acting as a chemoattractant via the microglia-specific P2Y12 receptor *in vitro* and *in vivo* (Haynes et al., 2006). Neurons and astrocytes can release ATP through exocytic and non-exocytic mechanisms (Illes et al., 2019), promoting the recruitment of microglial processes to facilitate functional interactions. P2Y12 mediates microglial process recruitment to neuronal somas in an activity dependent manner, as observed during stroke or seizures (Eyo et al., 2014; Cserép et al., 2020). In the context of these metabolic or hyper-excitatory insults, the recruitment appears to be neuroprotective; however, the mechanisms of this protection are not yet known. P2Y12 is also implicated in neurogenesis (Diaz-Aparicio et al., 2020), microglial rearrangements (Eyo et al., 2018), virus response (Fekete et al., 2018), and critical-period synaptic plasticity (Sipe et al., 2016). Thus, microglial P2Y12, originally described as a chemotactic receptor, has diverse functions in the nervous system.

However, little is known about the intracellular effectors downstream of P2Y12 activation. Many chemoattractant receptors, including P2Y12, are G_i_-coupled GPCRs, and chemotactic signaling pathways are often driven by the βγ subunit of the G_i_-protein heterotrimer (Gβγ; Haastert and Devreotes, 2004). Gβγ activates diverse targets (Lin and Smrcka, 2011), including phosphoinositide-3-kinase γ (PI3Kγ), which translates an extracellular chemoattractant gradient into spatially biased intracellular signaling. PI3Kγ phosphorylates phosphatidylinositol 4,5-bisphosphate (PIP_2_), a membrane phospholipid, forming phosphatidylinositol (3,4,5)-trisphosphate (PIP_3_; Vanhaesebroeck et al., 2012). PIP_3_ then activates other proteins to induce directional cellular motility and chemotaxis (Devreotes and Horwitz, 2015). PI3Kγ is unique among the Class I PI3Ks in that it is activated by Gβγ, whereas PI3Kα, PI3Kβ, and PI3Kδ require activation by receptor tyrosine kinases (RTKs; Vanhaesebroeck et al., 2012). Thus, it is a promising candidate to mediate P2Y12-dependent chemotaxis and directed microglial motility.

While Gβγ-PI3Kγ signaling has been well studied in the context of chemotaxis in the peripheral immune system, few studies have examined its role in ATP-dependent chemotaxis in microglia. In the CNS, PI3Kγ expression is highly enriched in microglia compared with other cell types (Zhang et al., 2014; He et al., 2018; Vanlandewijck et al., 2018; Zeisel et al., 2018). Studies of whole-cell microglial chemotaxis *in vitro* suggest that ATP-dependent chemotaxis is mediated, in part, by PI3K (Ohsawa et al., 2007; Irino et al., 2008). However, cell culture conditions affect both the microglial phenotype (Butovsky et al., 2014) and the cellular mechanisms underlying chemotaxis (Yamada and Sixt, 2019), so direct comparisons to *in vivo* function are difficult. In an *ex vivo* acute slice preparation, in which microglial morphology and ATP-dependent response is largely preserved, pan-inhibition of PI3K reduces the ability of microglial processes to respond to focal ATP release (Wu et al., 2007), although the role of PI3Kγ in particular was not addressed.

In this study, we sought to definitively determine whether PI3Kγ is required as a potential downstream effector of P2Y12 in both ATP-mediated directed microglial motility and experience-dependent synaptic plasticity. First, we characterized the morphology and homeostatic surveillance of microglia in PI3Kγ-deficient mice. We then used a combination of pharmacology and transgenic studies in an acute slice preparation to demonstrate that PI3Kγ is not a predominant mediator of the microglial response to focal damage or ATP-release. It is important to note that the absence or inhibition of PI3Kγ had an intermediate effect on the coordinated movement of microglial processes toward the source of ATP, suggesting that PI3Kγ may be playing a modulatory role. We found similar results *in vivo* where the gross response to focal injury was largely preserved but subtle deficits in long-range process motility were observed. Finally, we found that critical-period ocular dominance plasticity in the visual cortex was largely unaffected in PI3Kγ knock-out (KO) mice. Altogether, our work suggests that PI3Kγ is not a major effector of P2Y12 activation in microglial roles in directed motility and synaptic remodeling.

## Materials and Methods

### Animals

All animal protocols were approved by the Institutional Animal Care and Use Committee at the University of Rochester Medical Center and conform to NIH guidelines. Male and female mice between the ages postnatal day (P)21–P120 were used for all experiments. All animals were bred in-house under standard 12/12 h light/dark cycle and fed *ad libitum* with standard chow. All mice are on a C57/BL6 background. P2Y12 KO and PI3Kγ KO mice were provided by the Nedergaard laboratory (Haynes et al., 2006) and the Wymann laboratory (Hirsch et al., 2000), respectively. To visualize microglia in acute slices and *in vivo*, Cx3cr1-EGFP/+ heterozygous mice (Jung et al., 2000; JAX 005582) were used and crossed with KO strains where indicated. To selectively delete PI3Kγ in microglia, the Cx3cr1-CreERT2 line (Parkhurst et al., 2013; JAX 021160) was crossed with floxed PI3Kγ mouse (PI3Kγ fl/fl), provided by the Wymann laboratory (Breasson et al., 2017).

### Histology

Experiments were conducted on wild-type, P2Y12 KO, and PI3Kγ KO mice during the visual critical period (P30–P34). Mice were anesthetized with a lethal dose of sodium pentobarbital. Whole brains were collected following transcardial perfusion and overnight postfixation with 4% paraformaldehyde solution (4°C). After dehydration in 30% sucrose (w/v), 50-μm coronal sections were obtained using a freezing microtome (Microm; Global Medical Instrumentation). Sections were processed free-floating at room temperature (RT). To prevent non-specific antibody and antigen binding, sections were blocked in peroxidase and bovine serum albumin (BSA) solutions. Sections were incubated overnight in a primary antibody solution (4°C, rabbit anti-Iba-1, 1:2500, Wako #019-19741), followed by a secondary antibody solution (2 h, at RT, goat-anti-rabbit Alexa Fluor 594, 1:500, Invitrogen #A-11037), mounted, and coverslipped with Prolong Gold antifade reagent with DAPI (Invitrogen P36935). Imaging and analysis were conducted blind to genotype of the samples. For analysis of microglial density and distribution, Iba1-labeled microglia located in the primary visual cortex were identified and imaged on an Olympus BX51 epifluorescent microscope equipped with a SPOT pursuit camera (SPOT Imaging) using a 10× (NA = 0.3) objective lens (Olympus). For density analysis, the number of Iba1-positive microglia within a region of interest (ROI) was manually counted using the Cell Counter plugin in ImageJ/FIJI (https://imagej.net/Fiji). This number was then divided by the area of the ROI to generate density. For distribution analysis, the coordinates of each cell obtained from the Cell Counter were then used as the input in a custom MATLAB (MathWorks) script to find the nearest neighbor distance for each cell and then averaged for all cells in the ROI. The spacing index was then calculated as the square of the nearest neighbor multiplied by the density. Three to six slices of visual cortex were analyzed per animal and averaged such that one animal represents one data point. Cell density and nearest neighbor distribution were compared across genotypes using one-way ANOVA with Tukey’s *post hoc* multiple comparisons tests.

For analysis of ramification, microglia were imaged on a Nikon A1R HD confocal microscope. For each section, a 30-step z-stack encompassing the entirety of a few microglia was collected with a z-step of 0.375 μm, *xy*-pixel width of 0.12 μm with a 40× objective lens (Nikon Apochromat λ LWD; NA = 1.15). All image processing was performed in ImageJ/FIJI. Maximum Z-projections were generated, and microglia entirely encompassed in the stack were isolated. Images were then passed through a median filter (for despeckling) and a Gaussian filter (for smoothing), and then manually thresholded such that the fine processes were preserved. Small unconnected particles (<100 pixels), and staining artifacts, were removed using the Analyze Particles tool. To quantify the ramification of each microglia, Sholl analysis was then performed on each thresholded projection using ImageJ/FIJI. ∼15 microglia were analyzed from each animal and averaged such that one animal represents one data point. The maximum and total number of intersections were used to compare genotypes using one-way ANOVA with Tukey’s *post hoc* multiple comparisons tests.

### Chronic cranial window preparation

For anesthesia, a fentanyl cocktail containing fentanyl (0.05 mg/kg), midazolam (5.0 mg/kg), and dexmedetomidine (0.5 mg/kg) was administered intraperitoneally for cranial window procedures and two-photon *in vivo* imaging sessions. During surgery and imaging, body temperature was maintained at 37°C using a heating pad and rectal temperature probe and a lubricating eye ointment was used to prevent eye drying. Aseptic technique was used throughout the surgery, and tools were sterilized in between surgeries using a bead sterilizer. Mice were mounted to a stereotaxic frame and head fixed. The scalp was removed, and the skull exposed and cleared of debris. A 3-mm biopsy punch was used to mark the skull over left V1. A craniotomy was performed using a dental drill and a 0.5-mm drill bit (Fine Science Tools) to drill through the skull, tracing the marking, while frequently irrigating with sterile saline. The window, consisting of a circular 5-mm glass cover slip glued to a 3-mm glass coverslip (Warner Instruments) with UV glue (Norland Optical Adhesive) was placed (3 mm side down) over the exposed dura. The window, surrounding skull, and incision site were sealed with C&B Metabond dental cement (Parkell). A custom headplate (emachineshop.com; design courtesy of the Mriganka Sur lab, MIT) was attached with dental cement. Slow-release buprenorphine (5 mg/kg, sc) was administered after surgery, and mice were monitored for 72 h postoperative. Mice were imaged at least two weeks after surgery. For imaging, mice were anesthetized with fentanyl cocktail, head fixed onto a stage, and imaged for ∼1 h with body temperature maintained at 37°C.

### Acute slice preparation

For all slice experiments, slices were imaged between 30 min to 3 h from the time of slicing to avoid the overt microglial activation (Gyoneva and Traynelis, 2013). For focal laser injury experiments in acute slices, mice were decapitated and the brain was rapidly removed into ice-cold bicarbonate-buffered artificial cerebrospinal fluid, containing the following (obtained from Fisher): 126 mm NaCl, 2.5 mm KCl, 1.25 mm KH_2_PO_4_, 10 mm glucose, 1.3 mm MgSO_4_, 26 mm NaHCO_3_, and 2.5 mm CaCl_2_; constantly bubbled with 95% O_2_, 5% CO_2_ (bicarb-ACSF; ∼297 mOsm). Coronal sections 400-μm thickness encompassing V1 were prepared using a vibratome (Vibratome 1000), with the brain submerged in ice-cold bicarb-ACSF. Slices were transferred to a recovery chamber containing RT bicarb-ACSF. Bicarb-ACSF (heated to 34–36°C) was also used as the extracellular solution during imaging. Slices for focal ATP release experiments were performed in a similar manner, with a few modifications: ice-cold sucrose-based slicing solution was used for initial submersion of the brain and for slicing, containing the following: 76 mm NaCl, 75 mm sucrose, 2.5 mm KCl, 1.25 mm NaH_2_PO_4_, 25 mm glucose, 7 mm MgCl_2_, 26 mm NaHCO_3_, and 0.5 mm CaCl_2_; bubbled with 95% O_2_, 5% CO_2_ (sucrose-ACSF; ∼305 mOsm). Slices were recovered and imaged in HEPES-buffered ACSF, containing the following: 140 mm NaCl, 2.5 mm KCl, 1 mm NaH_2_PO_4_, 10 mm glucose, 2 mm MgCl_2_, 10 mm HEPES, and 2 mm CaCl_2_; pH adjusted to 7.4 with 5 M NaOH; and bubbled with 100% O_2_ (HEPES-ACSF; ∼297 mOsm). HEPES-ACSF was used in these ATP puff experiments as extracellular solution to precisely control the pH within the ATP filled pipet, as recommended by (Kyrargyri et al., 2019). For imaging, slices were placed in a perfusion chamber (RC-27L, Warner Instruments), continuously perfused with ACSF at a rate of ∼2 ml/min and imaged at a depth of ∼50–100 μm to avoid areas damaged from the slicing procedure (Eyo et al., 2014; Kyrargyri et al., 2019).

### Pharmacological reagents

For slice experiments, all drugs were solubilized in DMSO (Sigma) and subsequently diluted 1000-fold in ACSF for a final concentration of 0.1% DMSO, which was used as vehicle control. Elinogrel (Oestreich, 2010), Cycholasin D (Krucker et al., 2000), and forskolin (Swiatkowski et al., 2016) were obtained from Tocris, IPI-549 (Evans et al., 2016; Henau et al., 2016) from SelleckChem, and Wortmannin (Wu et al., 2007) from Sigma. Drugs were bath-applied through the perfusate solution, including a 10- to 15-min pretreatment before imaging.

### Two-photon imaging

A custom built two-photon laser scanning microscope (Ti:Sapphire laser, Mai-Tai, Spectra physics; Fluoview confocal scan head, BX61 microscope frame, 20× 0.95 NA water-immersion objective, Olympus) was used to image cortical EGFP+ microglia *in vivo* and in acute brain slices. Excitation was achieved with 920 nm (100 fs pulse at 80 MHz) and emission was collected through a 580/180 bandpass filter. In ATP-puff experiments, a 565 dichroic mirror was used to separate emission from EGFP (580/180 filter) and rhodamine (578/108 filter). Z-stacks were acquired at 4× digital zoom (XY pixel distance of 0.25 μm) with z-step size of 1 or 2 μm. For time lapse imaging, z-stacks were acquired at 1- or 2-min intervals. Image analysis was performed blind to genotype/treatment. The image analysis pipeline consisted of several preprocessing steps in ImageJ followed by thresholding and analysis in MATLAB. Preprocessing in ImageJ consisted of the following consecutive steps: (1) 3D registration to correct for motion using the 3DCorrect plugin (Parslow et al., 2014); (2) Maximum Z-projections encompassing 20 μm of tissue in the z-direction in slices and 10 μm *in vivo* (the higher density of microglial processes *in vivo* necessitated a smaller volume) to generate time-lapse movies; (3) Despeckling with a median filter; (4) a second registration step using the MultiStackReg plug in (bradbusse.net); and (5) bleach correction using the histogram matching method. Only the generation of Maximum Z-projections (2) requires user input. The resulting “xyt” time series were used for analysis with custom MATLAB scripts. ImageJ macros for batch processing and MATLAB scripts are available on GitHub (https://github.com/majewska-lab).

#### Motility and surveillance

Cortical microglia were imaged through a cranial window. Z-stacks were taken every 2 min for 32 min. The principles behind our analysis of microglial motility and surveillance have been previously described, by our lab (Sipe et al., 2016) and others (Madry et al., 2018; Bernier et al., 2019). New MATLAB scripts were developed for this paper to streamline the analysis. For each time series image, a threshold was manually selected to include fine processes while excluding background noise. Binarized images of consecutive time points were then overlaid. Positive pixels were classified as either stable (present in both time points, white), extensions (only present in second time point, green), or retractions (only present in first time point, magenta). The motility index was calculated as the sum of extended and retracted pixels divided by the stable pixels, averaged over the course of the imaging session. To calculate surveillance, or the total area surveyed by microglia across the imaging session, the binarized “xyt” image was maximum-projected in the “t” dimension, and the fraction of pixels that are positive is determined.

#### Laser injury

Laser injuries were induced by tuning the laser to 780 nm, increasing the power ∼5-fold, and running a point scan for 10–15 s. This generated an auto-fluorescent core indicative of tissue damage. After induction of the laser injury, z-stacks were taken every 2 min, for 30 min. For *in vivo* laser injury experiments, z-stacks were taken every 2 min for 62 min.

#### Focal ATP release

A 1 mm ATP (Sigma), 4% tetramethylrhodamine (“rhodamine”; average molecular weight 4400; Sigma) solution was prepared in HEPES-ACSF and backfilled into a glass microelectrode (∼3–4 MΩ resistance; BF150-110-10 glass pulled with P-97 puller, Sutter). The glass microelectrode was slowly inserted into cortical tissue using a micromanipulator (MP225, Sutter). The end of the pipet was connected to a Picospritzer III (Parker). An initial z-stack was obtained, then a pressure pulse (100 ms, 15 psi) released ATP onto the slice during the second time frame (*t* = 0 min). Additional z-stacks were acquired for 30 min (every 1 or 2 min).

### Image analysis

#### Analysis of laser ablation response

In this study, we have analyzed two aspects of the microglial chemotactic response: the convergence of microglial processes on a central source of ATP, and the coordinated movement of process toward the source. Our measurement of convergence around the laser injury is conceptually similar to what has been previously published (Davalos et al., 2005; Sipe et al., 2016). Images were binarized as described above. A polygon was outlined around the auto-fluorescent core generated by the laser injury, and this area was excluded from analysis. A donut shaped ROI of thickness 40 pixels (10 μm) was generated around the central core, or center. Convergence was calculated as the fraction of this ROI that was occupied by positive pixels, subtracted by the initial occupancy. A negative value for convergence is indicative of fluorescent debris that has disappeared and has not been replaced by microglial processes. To make statistical comparisons across groups, the convergence at 30 min (and 62 min for *in vivo* samples) was used. While this is a useful measurement of the microglial process accumulation in the ROI, it does not capture any information about the microglial response outside of the ROI, including directional movement. In order to capture movement throughout the field of view, we used an optic flow-based approach. The “xyt” time series was used to generate optic flow vectors by the method of Farneback (Farnebäck, 2003; MATLAB image processing toolbox). This method compares two consecutive frames and determines, for each pixel, the direction and magnitude of motion in the image (Extended Data Fig. 1-1). To find the degree to which this movement was directed toward the site of injury, the velocity vectors were projected onto the normalized vector pointing toward the injury, generating a measurement of directional velocity. This produced positive numbers for movement toward the injury, and negative numbers for movement away from the injury. In order to isolate microglial movement, a binary mask based on the thresholded images was applied to the vectors. Finally, the average directional velocity was calculated across all microglial pixels. This value is reported as arbitrary units (a.u.). For slice experiments, in which there is limited long-range motility, this analysis was restricted to within 200 pixels (50 μm) of the central injury. For the *in vivo* experiments, we conducted additional analyses on longer-range motility (200–300 pixels; 50–75 μm). In order to compare across groups, the average directional velocity from 10 to 20 min was used. This excluded initial artifacts caused by tissue deformation as well as the period after convergence has largely completed. To examine the spatiotemporal dynamics of the *in vivo* injury response, we calculated the directional velocity as a function of distance from the center, binning the radii in increments of 5 μm (Extended Data Fig. 6-1). For each “xyt” image, this analysis protocol requires the user to select a threshold and to manually outline the central injury. Beyond that, it requires no further user input.

**Figure 1.**
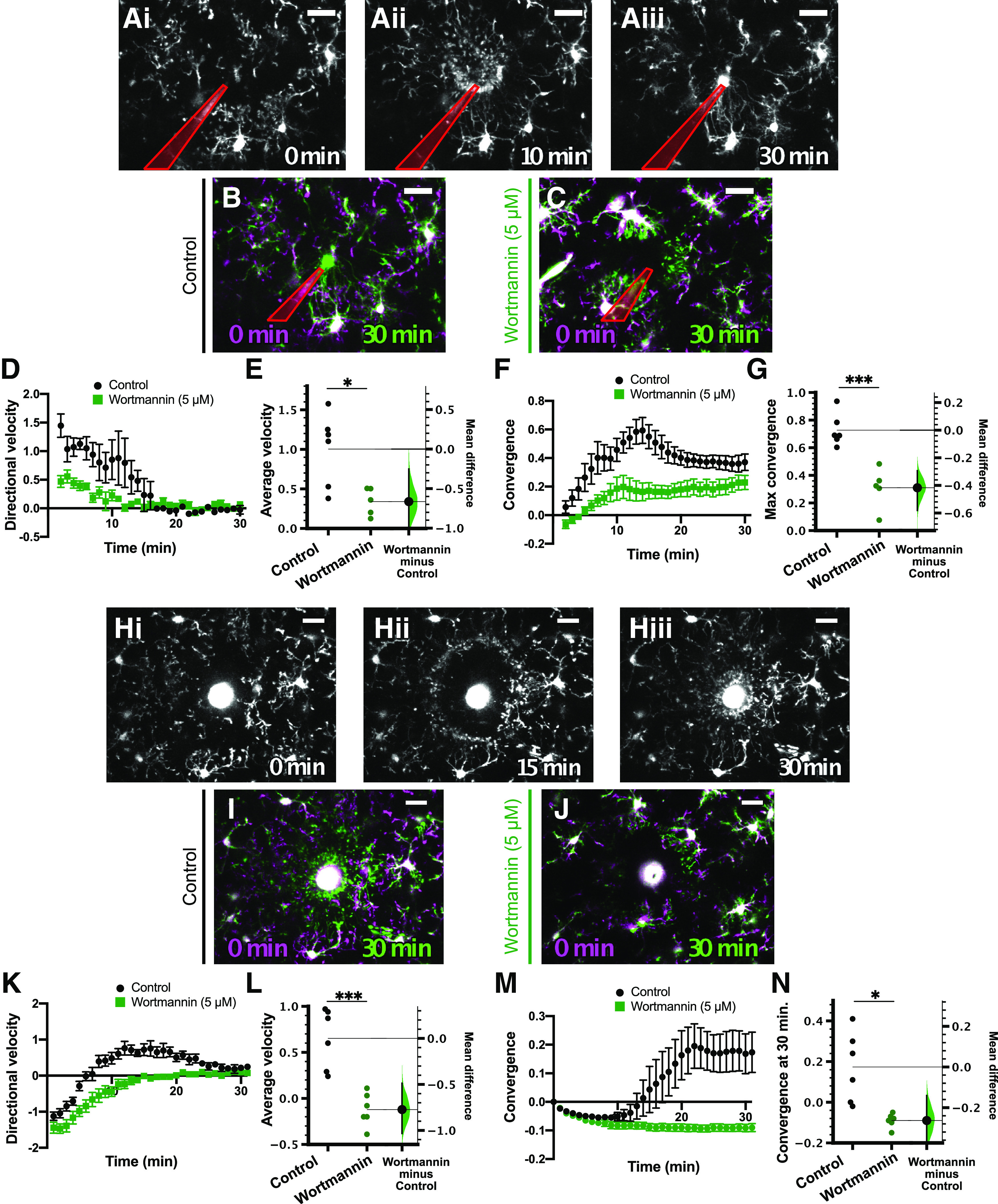
Pan-PI3K inhibition blocked the microglial response to focal laser injury in acute cortical slices. ***A***, Representative images of the microglial response to focal ATP release in a control (0.1% DMSO vehicle) slice at 0 min (***Ai***), 10 min (***Aii***), and 30 min (***Aii***) after ATP puff. ***B***, ***C***, Overlays of representative responses from control (***B***, overlay of ***Ai*** and ***Aiii***), 5 μm wortmannin-treated (pan-PI3K inhibitor; ***C***) slices at the indicated time points. Images at later time points (green) are overlaid with initial images (*t* = 0 min, magenta). In ***A–C***, the pipette is outlined in red. ***D***, Time course of directional velocity, with one frame acquired every 1 min. Quantification begins at *t* = 2 min (two frames after puff) to avoid artifacts from the dye included in the pipette solution which leaks into the imaging field at earlier time points. ***E***, The mean difference between control and Wortmannin-treated groups is shown in the Gardner–Altman estimation plot. Both groups are plotted on the left axes; the mean difference is plotted on a floating axes on the right as a bootstrap sampling distribution. The mean difference is depicted as a dot; the 95%CI is indicated by the ends of the vertical error bar. Wortmannin significantly blocked the directional response [comparisons made on average velocity calculated in the interval of 2–10 min after ATP presentation; unpaired *t* test, *p* = 0.014; unpaired mean difference is −0.665 (95%CI −0.991, −0.253)]. ***F***, Time course of convergence. Maximum convergence was used for statistical comparisons because microglial processes enter the pipet tip after convergence, decreasing this value. ***G***, Wortmannin reduces the maximum convergence as shown in the Gardner–Altman plot [unpaired *t* test, *p* = 0.0006; unpaired mean difference is −0.417 (95%CI −0.582, −0.294)]. ***H***, Representative images of the microglial response to laser injury (bright circular feature in the center of image) in a control cortical slice immediately after injury (***Hi***; 0 min), as the microglial processes moved toward the injury site (***Hii***; 15 min), and after the microglial processes converged on the injury site (***Hiii***; 30 min). ***I***, Overlay of images of the microglial response at 0 min (***Hi***; magenta) and at 30 min (***Hiii***; green) demonstrating convergence of processes around the injury after 30 min. ***J***, Similar overlay showing the microglial response to laser injury in a wortmannin-treated (5 μm) slice at 0 min (magenta) and 30 min (green) after the injury. Microglial processes did not converge around injury as shown by the lack of green pixels around the core. ***K***, Time course of the directional velocity in control (black circles) and wortmannin-treated (green squares) slices showing that wortmannin substantially decreased the motility directed toward the ablation core. Note that the negative directional velocity that occurs soon after ablation is an artifact of tissue deformation following the laser injury. ***L***, Comparison of the average velocity from 10 to 20 min (encompassing the peak of the response) showed significant decrease in the wortmannin-treated group [unpaired *t* test; *p* = 0.0005; unpaired mean difference is −0.773 (95%CI −1.03, −0.487)]. ***M***, Time course of convergence in control and wortmannin-treated slices. ***N***, Comparison of the convergence at 30 min showed a significant decrease in the wortmannin-treated group [Welch’s *t* test; *p* = 0.013; unpaired mean difference is −0.263 (95%CI −0.4, −0.14)]. *n* = 6 control slices, 5 wortmannin-treated slices; from 6 animals total for focal ATP release experiments. *n* = 6 control slices, 6 wortmannin-treated slices; from 6 animals total for laser injury experiments; *p < 0.05, **p < 0.01, ***p < 0.001. Data are presented as mean ± SEM. Scale bar: 20 μm. Additional information on the calculation of directional velocity is provided in Extended Data Figure 1-1 and Movie 9.

10.1523/ENEURO.0311-20.2020.f1-1 Extended Data Figure 1-1 Schematic description of directional velocity calculations. ***A***, For each pixel in the image, a velocity vector (u,v) is calculated representing the movement of that pixel from one time point to the next using an optic flow function in MATLAB. ***B***, To calculate the component of that movement that is directed towards the injury site or chemoattractant source, the velocity vector (u,v) is projected onto the relative position vector (x_rel_,y_rel_), the vector pointing towards the center of the injury. At this point, the binarized microglial image is used to restrict analysis to only those vectors originating from pixels that represent microglia. ***C***, The magnitude and sign (positive = towards, green; negative = away, red) of these vectors are averaged to generate a measure of “directional velocity.” ***D–F***, Representative microglial response *in vivo* to focal laser injury. Quiver plots have been used to overlay a subset of the velocity vectors (u,v) on the images, with the size of the arrows proportional to the magnitude of the velocity, and the color corresponding to the sign of the directional component (as in ***C***). Vectors were computed for pixels within 75 μm of the injury center. ***D***, Initial image at *t* = 0 min. There are no vectors calculated at this point. ***E***, Response at 14 min, showing coordinated movement of a ring of microglial processes converging on the injury. ***F***, Response at 30 min, where the microglial processes have converged on the core and the directed movement is less pronounced. More diffuse movement towards the injury site is still present. ***G***, Time course of average directional velocity for the experiment shown in ***D–F*** (black line, circles). Also plotted is the average directional velocity of a time lapse capturing baseline surveillance with no injury from a different imaging session (magenta, squares), demonstrating that there is no directional velocity in the absence of a focal injury. A similarly-sized polygon was generated at the center of the image to generate the ROI used in the analysis. Scale bar: 20 μm. Download Figure 1-1, EPS file.

#### Analysis of directed motility to ATP

To analyze the microglial response to ATP puff, the protocol was slightly modified. Instead of a drawing a polygon around the central injury, a point was placed at the end of the pipet tip. Next, a polygon was drawn around the pipet, to exclude it from the analysis. For convergence, a circular ROI of radius 50 pixels (12.5 μm) centered on the tip of the pipet was used as the area in which to calculate microglial occupancy, instead of the donut ROI around the injury core. Over time, microglial processes entered the pipet tip, thus leading to a decreased value of convergence. Thus, to compare across groups, we used the maximum convergence.

For directional velocity, the pipet tip was used as the center. The circular ROI used for convergence was excluded from the directional velocity analysis because of artifacts caused by movement at the tip of the pipet. Quantification of microglial response begins two frames after ATP release (*t* = 4 min at one frame/2 min; *t* = 2 min at one frame/1 min) to avoid artifacts from the dye included in the pipette solution which leaks into the imaging field at earlier time points. For comparisons between groups, the average directional velocity from 2 to 10 min (4–10 min when z-stacks were obtained every 2 min) was calculated, encompassing the bulk of the microglial response. After the processes converge and clog the pipet, directional velocity falls to zero.

### Microglial-specific PI3Kγ deletion

Cx3cr1-CreERT2 mice (referred to as Cx3cr1-Cre; Parkhurst et al., 2013) were crossed with PI3Kγ fl/fl (Breasson et al., 2017) mice to generate the tamoxifen inducible microglial deletion of PI3Kγ. Controls used for these experiments were tamoxifen-treated Cx3cr1-Cre/+ heterozygous mice. Tamoxifen (50 μg; Sigma) solubilized in corn oil (2 mg/ml) was administered on three consecutive days to pups aged ∼P2–P4 via oral gavage. Tamoxifen induction of Cre activity was confirmed using fluorescence-assisted cell sorting (FACS) to isolate and genotype microglia. Tamoxifen-treated mice aged P30–P35 were given an overdose of sodium pentobarbital and transcardially perfused with ice cold 0.15 m phosphate buffer (PB). The brain was rapidly removed and placed in ice cold FACS buffer (0.5% BSA, Sigma A2153; PBS without Ca or Mg, Invitrogen 20012-027). Samples were placed on ice for the remainder of the preparation. The cortices were dissected and homogenized manually using a Dounce homogenizer. The homogenate was filtered through a 70-μm cell strainer (Fisher) and centrifuged at 210 × *g* for 7 min. at 4°C. The supernatant was discarded, the pellet resuspended in FACS buffer, and Myelin Removal Beads II (Miltenyi) were added. After a 15-min incubation, the solution was passed through a 70-μm filter and then primed magnetic LS columns (Miltenyi). The resulting myelin-depleted suspension was centrifuged and re-suspended. An Fc Block (Biolegend) was added for 15 min. The sample was then labeled with anti-CD11b-BV786 (1:200, BD, clone M1/70), anti-CD45-APC (1:400, BD, clone 30-F11) for 30 min in the dark. After centrifugation and re-suspension, propidium iodide (PI; 10 μg/ml) was added just before sorting to label dead cells. The following compensation controls were included: unstained cell suspension, anti-CD11b-BV786 beads, anti-CD45-APC beads (eBiosciences, 01-11142), and Triton X-100-treated dead cell suspension (for PI). Samples were run on an 18-color FACSAria II flow cytometer. Microglial cells were identified as CD11b+, CD45lo, PI– (Extended Data Fig. 7-1). Sorted microglia were collected into PBS for DNA isolation using the Qiagen DNAeasy DNA isolation kit. Cx3cr1-Cre/+; PI3Kγ fl/fl mice (PI3Kγ cKO) from three separate litters were included in our confirmation experiments. Sorted microglia from Cx3cr1-Cre/+; PI3Kγ wild-type (WT), and PI3Kγ fl/fl mice were used as additional controls. Two separate PCR reactions were run on these samples: (1) PCR for the WT (332-bp product) and the floxed PI3Kγ allele (476-bp product; forward, ACACCCAACCCAGAACCAAC; reverse, AAGGGGAGAAGGGAGAGGTG); and (2) PCR for the excision product (635 bp; forward, ACACCCAACCCAGAACCAAC; reverse, CCATGTGTGAAGGTGACATACATT), only present after Cre-mediated excision of the floxed PI3Kγ allele (Extended Data Fig. 7-1). Overall, we observed complete excision of the PI3Kγ floxed allele in microglial samples from three separate tamoxifen-treated PI3Kγ fl/fl; Cx3cr1-Cre/+ litters, as determined by no visible PI3Kγ fl/fl allele and presence of the excision product.

### Monocular deprivation

Animals in the peak critical period for ocular dominance plasticity (P26–P29; Gordon and Stryker, 1996) were assigned to non-deprived (ND) or 4 d of monocular deprivation (4D MD) cohorts. For 4D MD, animals were anesthetized with isoflurane (5% induction; 2% maintenance), and given carprofen analgesic before surgery (5 mg/kg). The margins of the right eyelids were removed, and Tobradex antibiotic eye ointment was applied to the eye and margins. The right eyelids were sutured together, and the mice quickly recovered. Before use in intrinsic optical signal (IOS) experiments, the eyelid sutures were carefully examined under a dissection scope. If there was any opening in the suture or visible damage to the eye, the mice were excluded.

### IOS imaging

Two sets of animals were used for these experiments (1) C57/BL6 and PI3Kγ KO mice to determine the effect of global PI3Kγ KO; and (2) Cx3cr1-Cre/+ and Cx3cr1-Cre/+; PI3Kγ fl/fl mice to determine the effect of microglial-specific PI3Kγ KO. Animals were anesthetized with isoflurane and dosed with chlorprothixene (2 mg/kg). The sutures were re-opened and the remaining eyelid margins were trimmed. Eyes were covered with clear silicone oil to prevent drying. The scalp was removed and skull cleared of membranes and debris. A flat metal bar was glued onto the right hemisphere, to head-fix the animal, leaving the left hemisphere uncovered. A 0.5% agarose solution was placed over the skull and sealed with a coverslip. Isoflurane was maintained at 0.75% during imaging. A custom built IOS imaging rig was used to measure the blood oxygenation level-dependent (BOLD) response of the left visual cortex to a periodic stimulus, as described in (Kalatsky and Stryker, 2003). A camera (DALSA 2M30 CCD) captured light reflected off the skull: 550-nm light for the vasculature and 700-nm light for the intrinsic signal. A horizontal bar drifting vertically (90° or 270°) was presented to the mice on an LCD screen located 30 cm from the mouse eyes. The stimulus frequency was 0.1 Hz, presented over 6 min. Each eye was imaged separately with stimulus in both directions. The normalized amplitude maps of the fast-Fourier transform of the intrinsic signal was averaged from both stimulus directions. The ipsilateral (left eye) and contralateral (right eye) maps were compared offline in MATLAB. The ocular dominance index (ODI) was calculated using the equation: ODI = (average contralateral response – average ipsilateral response)/(average contralateral response + average ipsilateral response).

### Statistical analysis

Statistical comparisons were made between groups using Prism 8 software (GraphPad). Sample size was not predetermined but is consistent with previously published studies (Sipe et al., 2016; Madry et al., 2018). All analyses were performed blinded to condition. Individual data points (“*n*”) represent individual mice, with the exception of drug treatment experiments in acute brain slices. For those, *n* represents individual slices. Multiple slices were used from each animal (no more than three). However, controls were interleaved within the treated groups such that no two data points for a given condition came from the same animal. For all analyses, α = 0.05. Statistical comparisons were done using un-paired two-tailed *t* tests, Welch’s *t* test (for samples with unequal variance), one-way ANOVA with Tukey’s *post hoc* comparisons, and two-way ANOVA with Sidak’s multiple comparisons, as indicated in the figure legends and statistics table, where *p* values are presented (Table 1). Data reported in the text are mean ± SEM.

**Table 1:** Statistics table

	Figure	Group comparison	Data structure	Statistical test	*p* value	Difference of means	95%CI
a	1*E*	Control vs wortmannin (5 μm)	Normal	Unpaired *t* test	*p* = 0.0139; *t* = 3.046; df = 9	−0.6647	−1.158 to −0.1711
	1*G*	Control vs wortmannin (5 μm)	Normal	Unpaired *t* test	*p* = 0.0006; *t* = 5.216; df = 9	−0.4174	−0.5984 to −0.2364
b	1*L*	Control vs wortmannin (5 μm)	Normal	Unpaired *t* test	*p* = 0.0005; *t* = 5.071; df = 10	−0.7733	−1.113 to −0.4335
	1*N*	Control vs wortmannin (5 μm)	Normal (unequal variance)	Welch’s *t* test	*p* = 0.0125; *t* = 3.679; df = 5.391	−0.2633	−0.4434 to −0.08326
c	2*B*	Density: genoytpe	Normal	One-way ANOVA	*p* = 0.1752; *F* = 1.912;		
		Control vs P2Y12 KO		Tukey’s *post hoc*	*p* = 0.1520	−74.57 microglia/mm^2^	−171.7 to 22.54
		Control vs PI3Kg KO		Tukey’s *post hoc*	*p* = 0.5830	−38.40 microglia/mm^2^	−135.5 to 58.71
		P2Y12 KO vs PI3Kg KO		Tukey’s *post hoc*	*p* = 0.5725	36.17 microglia/mm^2^	−53.74 to 126.1
d	2*C*	Spacing index: genotype	Normal	One-way ANOVA	*p* = 0.2995; *F* = 1.285		
		Control vs P2Y12 KO		Tukey’s *post hoc*	0.3074	−0.01460	−0.03911 to 0.009914
		Control vs PI3Kg KO		Tukey’s *post hoc*	0.4122	−0.01254	−0.03706 to 0.01197
		P2Y12 KO vs PI3Kg KO		Tukey’s *post hoc*	0.9714	0.002052	−0.02064 to 0.02474
e	2*F*	Max intersections: genotype	Normal	One-way ANOVA	*p* = 0.6724; *F* = 0.4064		
		Control vs P2Y12 KO		Tukey’s *post hoc*	0.7245	1.123	−2.608 to 4.854
		Control vs PI3Kg KO		Tukey’s *post hoc*	0.9955	0.1279	−3.504 to 3.760
		P2Y12 KO vs PI3Kg KO		Tukey’s *post hoc*	0.7234	−0.9952	−4.293 to 2.302
f	2*G*	Total intersections: genotype	Normal	One-way ANOVA	*p* = 0.1906; *F* = 1.830		
		Control vs P2Y12 KO		Tukey’s *post hoc*	0.2044	23.51	−10.28 to 57.29
		Control vs PI3Kg KO		Tukey’s *post hoc*	0.8639	6.634	−26.26 to 39.53
		P2Y12 KO vs PI3Kg KO		Tukey’s *post hoc*	0.3391	−16.87	−46.73 to 12.99
g	3*B*	Motility: genotype	Normal	One-way ANOVA	*p* = 0.0060; *F* = 7.174		
		Control vs P2Y12 KO		Tukey’s *post hoc*	0.1208	−0.04231	−0.09423 to 0.009605
		Control vs PI3Kg KO		Tukey’s *post hoc*	0.1032	0.04410	−0.007822 to 0.09601
		P2Y12 KO vs PI3Kg KO		Tukey’s *post hoc*	0.0043	0.08641	0.02754 to 0.1453
h	3*C*	Surveillance: genotype	Normal	One-way ANOVA	*p* = 0.0432; *F* = 3.847		
		Control vs P2Y12 KO		Tukey’s *post hoc*	0.2417	−0.02299	−0.05824 to 0.01225
		Control vs PI3Kg KO		Tukey’s *post hoc*	0.3364	0.01992	−0.01533 to 0.05516
		P2Y12 KO vs PI3Kg KO		Tukey’s *post hoc*	0.0345	0.04291	0.002947 to 0.08287
i	4*E*	Control vs elinogrel (2 μm)		Unpaired *t* test	*p* = 0.0005; *t* = 6.702; df = 6	−1.658	−2.263 to −1.053
	4*G*	Control vs elinogrel (2 μm)		Unpaired *t* test	*p*= 0.0048; *t* = 4.349; df = 6	−0.4734	−0.7398 to −0.2070
j	4*I*	Control vs IPI-549 (1 μm)		Unpaired *t* test	*p* = 0.0310; *t* = 2.692; df = 7	−0.5813	−1.092 to −0.07060
	4*K*	Control vs IPI-549 (1 μm)		Unpaired *t* test	*p* = 0.1468; *t* = 1.632; df =7	−0.1158	−0.2836 to 0.05201
k	4*M*	Average velocity: genotype	Normal	One-way ANOVA	*p* = 0.0003; *F* = 20.01		
		Control vs P2Y12 KO		Tukey’s *post hoc*	0.0002	0.9095	0.5138 to 1.305
		Control vs PI3Kg KO		Tukey’s *post hoc*	0.0089	0.5199	0.1445 to 0.8952
		P2Y12 KO vs PI3Kg KO		Tukey’s *post hoc*	0.0421	−0.3897	−0.7650 to −0.01428
l	4*N*	Max convergence: genotype	Normal	One-way ANOVA	*p* = 0.0011; *F* = 14.58		
		Control vs P2Y12 KO		Tukey’s *post hoc*	0.0010	0.5960	0.2858 to 0.9062
		Control vs PI3Kg KO		Tukey’s *post hoc*	0.2314	0.1891	−0.1052 to 0.4833
		P2Y12 KO vs PI3Kg KO		Tukey’s *post hoc*	0.0090	−0.4070	−0.7012 to −0.1127
m	5*E*	Average velocity: treatment	Normal	One-way ANOVA	*p* < 0.0001; *F* = 25.42		
		Control vs elinogrel (2 μm)		Tukey’s *post hoc*	<0.0001	0.8800	0.5745 to 1.186
		Control vs IPI-549 (1 μm)		Tukey’s *post hoc*	0.0014	0.4725	0.1743 to 0.7707
		elingorel (2 μm) vs IPI-549 (1 μm)		Tukey’s *post hoc*	0.0071	−0.4076	−0.7132 to −0.1020
n	5*G*	Convergence at 30 min: treatment	Normal	One-way ANOVA	*p* < 0.0001; *F* = 27.90		
		Control vs elinogrel (2 μm)		Tukey’s *post hoc*	<0.0001	0.1601	0.1070 to 0.2132
		Control vs IPI-549 (1 μm)		Tukey’s *post hoc*	0.0007	0.08762	0.03578 to 0.1395
		P2Y12 KO vs PI3Kg KO		Tukey’s *post hoc*	0.0059	−0.07247	−0.1256 to −0.01936
o	5*I*	Average velocity: genotype	Normal	One-way ANOVA	*p* = 0.0002; *F* = 14.05		
		Control vs P2Y12 KO		Tukey’s *post hoc*	0.0001	0.8565	0.4440 to 1.269
		Control vs PI3Kg KO		Tukey’s *post hoc*	0.0312	0.4502	0.03772 to 0.8627
		P2Y12 KO vs PI3Kg KO		Tukey’s *post hoc*	0.0540	−0.4063	−0.8187 to 0.006227
p	5*K*	Convergence at 30 min: genotype	Normal	One-way ANOVA	*p* = 0.0014; *F* = 9.695		
		Control vs P2Y12 KO		Tukey’s *post hoc*	0.0030	0.1074	0.03683 to 0.1780
		Control vs PI3Kg KO		Tukey’s *post hoc*	0.9884	0.004017	−0.06655 to 0.07459
		P2Y12 KO vs PI3Kg KO		Tukey’s *post hoc*	0.0041	−0.1034	−0.1739 to −0.03281
q	6*E*	Convergence at 30 min: genotype	Normal	One-way ANOVA	*p* = 0.0003; *F* = 12.08		
		Control vs P2Y12 KO		Tukey’s *post hoc*	0.0018	0.2634	0.09725 to 0.4296
		Control vs PI3Kg KO		Tukey’s *post hoc*	0.4782	−0.07407	−0.2324 to 0.08426
		P2Y12 KO vs PI3Kg KO		Tukey’s *post hoc*	0.0004	−0.3375	−0.5197 to −0.1553
r	6*F*	Convergence at 62 min: genotype	Normal	One-way ANOVA	*p* = 0.0553; *F* = 3.333		
		Control vs P2Y12 KO		Tukey’s *post hoc*	0.6003	0.05798	−0.09193 to 0.2079
		Control vs PI3Kg KO		Tukey’s *post hoc*	0.1753	−0.1054	−0.2482 to 0.03746
		P2Y12 KO vs PI3Kg KO		Tukey’s *post hoc*	0.0516	−0.1633	−0.3277 to 0.0009932
s	6*H*	Average velocity <50 μm 10–20 min: genotype	Normal	One-way ANOVA	*p* = 0.0625		
		Control vs P2Y12 KO		Tukey’s *post hoc*	0.0559	1.052	−0.02320 to 2.127
		Control vs PI3Kg KO		Tukey’s *post hoc*	0.3630	0.5656	−0.4587 to 1.590
		P2Y12 KO vs PI3Kg KO		Tukey’s *post hoc*	0.5605	−0.4864	−1.665 to 0.6923
t	6*I*	Average velocity <50 μm 34–62 min: genotype	Normal	One-way ANOVA	*p* < 0.0001; *F* = 16.37		
		Control vs P2Y12 KO		Tukey’s *post hoc*	<0.0001	−0.4057	−0.6056 to −0.2058
		Control vs PI3Kg KO		Tukey’s *post hoc*	0.9988	0.009845	−0.1806 to 0.2003
		P2Y12 KO vs PI3Kg KO		Tukey’s *post hoc*	0.0002	0.4155	0.1964 to 0.6347
u	6*K*	Average velocity 50–75 μm 10–20 min: genotype	Normal	One-way ANOVA	*p* < 0.0001; *F* = 17.51		
		Control vs P2Y12 KO		Tukey’s *post hoc*	0.0152	−0.4969	−0.9042 to −0.08957
		Control vs PI3Kg KO		Tukey’s *post hoc*	0.0051	0.5476	0.1595 to 0.9356
		P2Y12 KO vs PI3Kg KO		Tukey’s *post hoc*	<0.0001	1.044	0.5979 to 1.491
v	6*L*	Average velocity 50–75 μm 34–62 min: genotype	Normal	One-way ANOVA	*p* = 0.0003; *F* = 11.99		
		Control vs P2Y12 KO		Tukey’s *post hoc*	0.0359	−0.2130	−0.4133 to −0.01265
		Control vs PI3Kg KO		Tukey’s *post hoc*	0.0270	0.2130	0.02217 to 0.4038
		P2Y12 KO vs PI3Kg KO		Tukey’s *post hoc*	0.0002	0.4260	0.2064 to 0.6455
w	7*C*	Deprivation × genotype	Normal	Two-way ANOVA: interaction	*p* = 0.4505; *F*_(1,28)_ = 0.5858		
		Deprivation		Factor	*p* = 0.0005; *F*_(1,28)_ = 15.49		
		Genotype		Factor	*p* = 0.1040; *F*_(1,28)_ = 2.824		
						Predicted (LS) mean diff:	
		WT: ND vs 4D MD		Sidak’s *post hoc*	0.0079	0.2226	0.05521 to 0.3900
		PI3Kg KO: ND vs 4D MD		Sidak’s *post hoc*	0.0472	0.1501	0.001620 to 0.2986
x	7*D*	Deprivation × genotype	Normal	Two-way ANOVA: interaction	*p* = 0.3074; *F*_(1,20)_ = 1.097		
		Deprivation		Factor	*p* = 0.0084; *F*_(1,20)_ = 8.554		
		Genotype		Factor	*p* = 0.9364; *F*_(1,20)_ = 0.006		
						Predicted (LS) mean diff:	
		Cx3cr1-Cre/+: ND vs 4D mD			0.0275	0.2247	0.02332 to 0.4260
		Cx3cr1-Cre/+ PI3Kg fl/fl: ND vs 4D mD			0.3285	0.1062	−0.07880 to 0.2912
						Difference of means:	
y	4-1*B*	Control vs cytochalasin D (1 μm)	Normal (unequal variance)	Welch’s *t* test	*p* = 0.0339; *t* = 3.625; df = 3.118	−0.6931	−1.289 to −0.09735
	4-1*D*	Control vs cytochalasin D (1 μm)	Normal	Unpaired *t* test	*p* = 0.0002; *t* = 6.948; df = 7	−0.5704	−0.6801 to −0.3347
z	4-1*F*	Control vs cytochalasin D (1 μm)	Normal (unequal variance)	Welch’s *t* test	*p* = 0.0528; *t* = 4.039; df = 2.076	−1.047	−2.124 to 0.02993
	4-1*H*	Control vs cytochalasin D (1 μm)	Normal (unequal variance)	Welch’s *t* test	*p* = 0.0387; *t* = 4.727; df = 2.084	−0.5800	−1.088 to −0.07202
zz	4-2*B*	Control vs forskolin (10 μm)	Normal	Unpaired *t* test	*p* = 0.069; *t* = 2.146; df = 7	−0.3249	−0.6829 to 0.0331
	4-2*D*	Control vs forskolin (10 μm)	Normal	Unpaired *t* test	*p* = 0.896; *t* = 0.135; df = 7	0.0293	−0.4843 to 0.5430

To graphically illustrate effect sizes, we used estimation graphics (https://www.estimationstats.com/#/; Ho et al., 2019). When comparing between two groups, Gardner–Altman plots were used. On the left axes, the raw data from both groups were plotted. The mean difference between the two groups was plotted on a floating axes to the right as a bootstrap sampling distribution. The mean difference is depicted as a dot, while the 95% confidence interval (CI) is indicated by the vertical bar. For comparisons of three groups, the experimental groups were compared with a shared control and the mean differences were graphed using a Cumming estimation plot. The raw data are plotted on the upper axes, while the mean difference, 95%CI, and bootstrap sampling distribution are plotted on the lower axes. Graphs were created using the DABEST package in python (https://github.com/ACCLAB/DABEST-python).

The DABEST package uses a bootstrapping technique to generate a distribution of mean differences, and then calculates the average and the 95%CI for this distribution, which is graphically displayed and reported in the figure legends. Notably, this is distinct from the mean and CIs of the effect sizes calculated while doing the hypothesis testing mentioned above (e.g., *t* test, ANOVA) and reported in the statistics table (Table 1). These intervals are derived with the pooled standard error under the assumption of a normal distribution.

### Code availability

The code/software described in the paper is freely available online at https://github.com/majewska-lab. The code is available as Extended Data 1. This code was run on a Macbook pro (early 2015, Intel Core i5) running MacOS 10.14.

10.1523/ENEURO.0311-20.2020.ed1 Extended Data 1 Code accessibility: code used for image analysis. Description of each file is included in the attached document. Download Extended Data 1, ZIP file.

## Results

PI3Kγ is highly enriched in microglia in the CNS (Zhang et al., 2014; He et al., 2018; Vanlandewijck et al., 2018; Zeisel et al., 2018), and has been shown to be critical for neuroinflammatory function, phagocytosis and chemotaxis toward complement (Passos et al., 2010; Schmidt et al., 2013, 2016; Lima et al., 2015; Schneble et al., 2017). Its role in P2Y12-dependent ATP-mediated directed process motility, which is critical to a number of both physiological and pathologic responses of microglia (Haynes et al., 2006; Eyo et al., 2014; Sipe et al., 2016; Cserép et al., 2020), has not been explored. Previous studies have implicated the PI3K pathway in regulating microglial chemotaxis to purines (Ohsawa et al., 2007; Wu et al., 2007; Irino et al., 2008), making it all the more likely that PI3Kγ is an effector of P2Y12 signaling. Before specifically testing the role of PI3Kγ in this process, we first wanted to ensure that PI3K signaling was indeed required for P2Y12-dependent microglial directed motility. Using Cx3cr1-EGFP mice (Jung et al., 2000), in which microglia are fluorescently tagged with EGFP, we monitored the dynamics of the microglial response to focal sources of ATP (ATP-filled micropipette or focal laser injury; Fig. 1; Movie 1). In order to characterize the microglial response, we developed new analyses to capture two aspects of process motility: the overall directional movement of microglia toward the ATP source (Extended Data Fig. 1-1) and the convergence of microglial processes on the source (for details, see Materials and Methods). The directional velocity measurement, reported as a.u., allows for the analysis of movement farther from the site of convergence, allowing for a more complete characterization of microglial directed motility. The convergence describes the accumulation of microglial processes in a defined region around the ATP source, and is calculated as the fraction of this space occupied subtracted by the initial occupancy. Thus, if an ATP source is only able to recruit nearby processes, microglial convergence may be unimpaired, but the average directional velocity of processes will be decreased.

Movie 1. Related to Figure 1. Microglial response to focal ATP release and focal laser injury in a vehicle-treated (0.1% DMSO) and wortmannin-treated (5 μM) acute cortical slice. Scale bar: 20 μm. One frame per 1 min. 10.1523/ENEURO.0311-20.2020.video.1

To test the involvement of the PI3K pathway, we used the pan-PI3K inhibitor wortmannin (5 μm; Fig. 1). Slices were pretreated for 10 min with the inhibitor and the inhibitor was also present in the perfusing solution throughout the imaging sessions. Using the two measures outlined above, we found that the microglial response to focal ATP release was decreased by pan-PI3K inhibition [*n* (slices) = 6 control, 5 wortmannin; Fig. 1*A–G*], although there was still some degree of motility toward the pipette in the presence of wortmannin (Fig. 1*C*; Movie 1). The average directional velocity, calculated as the average from 2 to 10 min, in vehicle-treated controls (1.00 ± 0.19 a.u.) was significantly different from that of wortmannin-treated slices (0.34 ± 0.08 a.u.; Fig. 1*E*; Table 1a). Wortmannin also significantly decreased the maximum convergence of microglial processes on the pipette tip (control 0.73 ± 0.05; wortmannin 0.31 ± 0.07; Fig. 1*G*; Table 1a). These results were in agreement with the finding that wortmannin blocks the microglial response to ATP puff in hippocampal slices (Wu et al., 2007). Furthermore, the microglial response to focal laser injury was also abrogated by 5 μm wortmannin treatment [*n* (slices) = 6 control, 6 wortmannin-treated; Fig. 1*H–N*]. The average velocity, calculated as the average directional velocity from 10 to 20 min, was decreased by wortmannin (control 0.65 ± 0.13 a.u.; wortmannin −0.12 ± 0.07 a.u.; Fig. 1*L*; Table 1b). Similarly, the convergence at 30 min was also decreased by wortmannin (control 0.17 ± 0.07; wortmannin −0.09 ± 0.01; Fig. 1*N*; Table 1b). Thus, PI3K is likely activated downstream of P2Y12. Because PI3Kγ is the isoform activated by Gβγ, we further investigated how inhibiting or genetically deleting this protein affects microglial characteristics, directed motility, and contributions to synaptic plasticity.

### PI3Kγ KO does not affect microglial morphology or surveillance under homeostatic conditions

**Figure 2.**
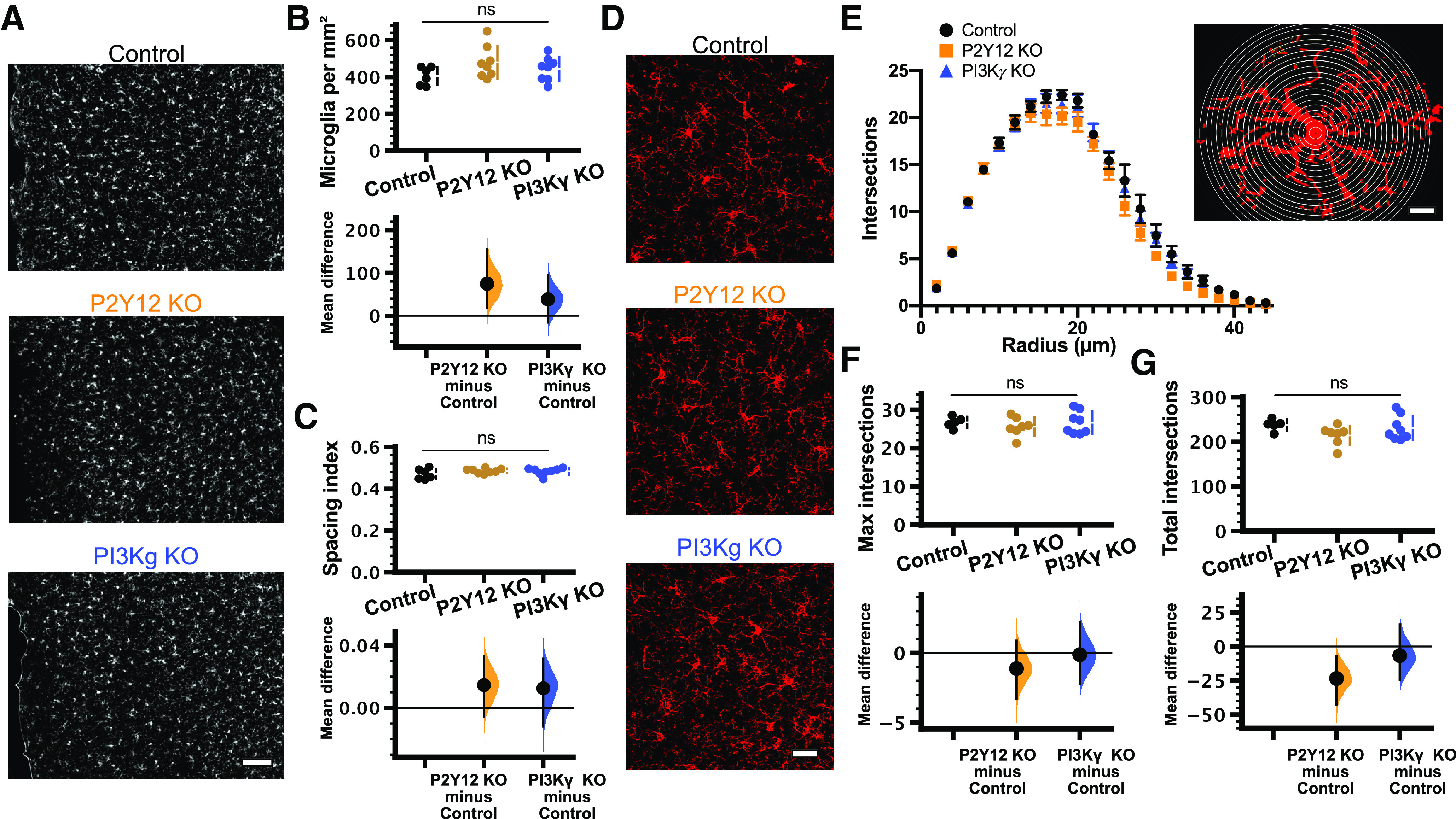
Microglial density, distribution, and morphology was unaffected in PI3Kγ KO mice. ***A***, Representative 10× epifluorescent images of Iba1-stained microglia in V1 from C57BL/6 control (top), P2Y12 KO (middle), and PI3Kγ KO (bottom) mice (scale bar: 100 μm). The mean difference for comparisons against the shared control are shown in the Cumming estimation plots. The raw data are plotted on the upper axes. On the lower axes, mean differences are plotted as bootstrap sampling distributions. Each mean difference is depicted as a dot. Each 95%CI is indicated by the ends of the vertical error bars. Analysis of the number and location of microglial cell bodies [*n* (animals) = 5 control; 7 P2Y12 KO; 8 PI3Kγ KO] revealed no differences in either cell density [***B***; one-way ANOVA *p* = 0.18; unpaired mean differences vs control: P2Y12 KO 74.6 (95%CI 17.7, 155); PI3Kγ KO 38.4 (95%CI −15.7, 94.1)] or the spacing index [***C***; one-way ANOVA *p* = 0.30; unpaired mean differences vs control: P2Y12 KO 0.0146 (95%CI −0.00574, 0.0334); PI3Kγ KO 0.0125 (95%CI −0.012, 0.0315)]. ***D***, Representative 40× confocal images of Iba1-stained microglia from the C57BL/6 control (top), P2Y12 KO (middle), and PI3Kγ KO (bottom) mice (scale bar: 30 μm). ***E–G***, Analysis of the branching complexity by Sholl analysis. ***E***, Plot of the number of intersections as a function of radius showed no apparent differences between genotypes [14–38 microglia per animal; *n* (animals) = 5 control; 7 P2Y12 KO; 8 PI3Kγ KO]. Inset, Schematic showing a representative microglial cell and the concentric rings (radius increasing in increments of 2 μm) used to calculate the number of processes as a function of distance from the soma (scale bar: 10 μm). Comparisons of the maximum number of intersections [***F***; one-way ANOVA *p* = 0.67; unpaired mean differences vs control: P2Y12 KO −1.12 (95%CI −3.27, 0.866); PI3Kγ KO −0.128 (95%CI −2.2, 2.22)] and the total intersections [***G***; one-way ANOVA *p* = 0.19; unpaired mean differences vs control: P2Y12 KO −23.5 (95%CI −42.6, −6.88); PI3Kγ KO −6.63 (95%CI −24.3, 16.2)] showed no differences between genotypes; ns, non-significant. Data are presented as mean ± SEM.

Despite the recent interest in PI3Kγ function in microglia, no studies have described how PI3Kγ loss impacts basal microglial morphology and distribution *in vivo*. Previous studies of PI3Kγ have been conducted either *in vitro* (Schmidt et al., 2013; Schneble et al., 2017) or in the context of pathology (Frister et al., 2014; Schmidt et al., 2016). Therefore, we first sought to characterize microglia under homeostatic conditions in the PI3Kγ KO mouse as changes in microglial number, distribution, morphology, and dynamics could affect microglial responses to ATP independently of P2Y12 signaling. Additionally, microglial morphology, as well as changes in microglial numbers and distribution are indicative of microglial activation or inflammatory state (Kettenmann et al., 2011), which could be affected by PI3Kγ loss. To determine whether loss of PI3Kγ phenocopies loss of P2Y12, we also included P2Y12 KO mice in our studies. We used immunohistochemical labeling of the microglial-specific marker Iba1 to investigate morphologic characteristics of microglia in fixed brain slices from healthy adolescent (P28–P34) mice [*n* (mice) = 6 control, 8 P2Y12 KO, 8 PI3Kγ KO; Fig. 2]. Similar to what has been previously reported for P2Y12 KO mice (Haynes et al., 2006), the density of microglia in binocular visual cortex was unchanged across genotypes (Fig. 2*B*; in microglia/mm^2^, 406 ± 20 for control; 480 ± 31 for P2Y12 KO; 444 ± 23 for PI3Kγ KO; Table 1c). Qualitatively, microglia seemed uniformly distributed throughout the cortex, and a nearest-neighbor analysis showed that the distribution of microglia throughout the cortex was not affected by the loss of PI3Kγ or P2Y12 (Fig. 2*C*; spacing index, 0.470 ± 0.010 for control; 0.485 ± 0.004 for P2Y12 KO; 0.483 ± 0.006 for PI3Kγ KO; Table 1d). To determine how PI3Kγ affects microglial morphology, we used high-resolution confocal microscopy to assess the branching complexity of microglia using Sholl analysis [*n* (mice) = 5 control, 7 P2Y12 KO, 8 PI3Kγ KO; Fig. 2*D–G*]. P2Y12 KO microglia showed a trend toward less ramification, as has been previously described (Sipe et al., 2016). A comparison of the maximum number of intersections (Fig. 2*F*; 26.73 ± 0.65 for control; 25.60 ± 0.93 for P2Y12 KO; 26.60 ± 1.04 for PI3Kγ KO; Table 1e) or total number of intersections (Fig. 2*G*; 238 ± 6 for control; 214 ± 8 for P2Y12 KO; 231 ± 10 for PI3Kγ KO; Table 1f) showed no differences across genotypes. The discrepancy between our results and those reported in Sipe et al., (2016) regarding the effects of P2Y12 KO on microglia morphology might be explained by slightly different imaging and analysis parameters. In this study, we were able to image very fine filopodial processes. If P2Y12 KO microglia have increased filopodial structures but decreased larger processes (possibly because of increased cAMP), increasing image resolution may lead to a normalization of the overall morphologic complexity as measured by Sholl analysis.

**Figure 3.**
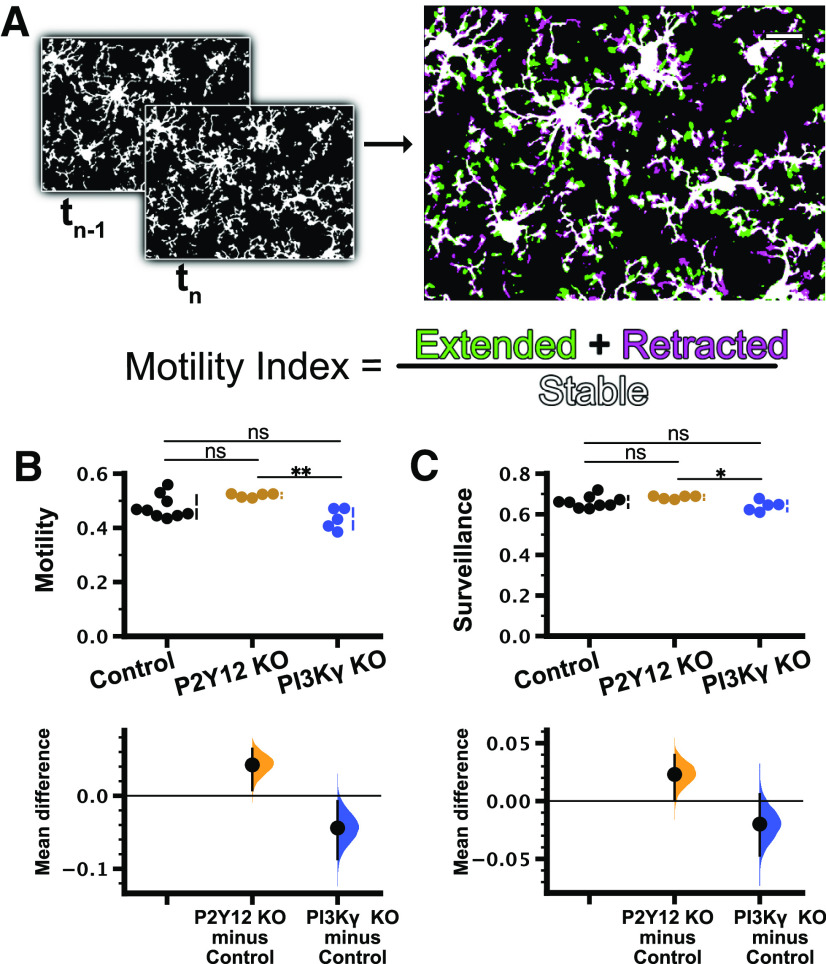
Microglial motility and surveillance were not affected in PI3Kγ KO mice. ***A***, Schematic illustrating the generation of the microglial motility index from time-lapse imaging (see Materials and Methods). ***B***, ***C***, Cumming estimation plots were used to show the mean differences between controls and experimental groups. ***B***, Motility in control mice was not significantly different from that in P2Y12 KO or PI3Kγ KO mice, but significant differences were observed between P2Y12 KO and PI3Kγ KO groups (one-way ANOVA, *p* = 0.0060; Tukey’s test: control vs P2Y12KO *p* = 0.12; control vs PI3Kγ KO *p* = 0.10; P2Y12 KO vs PI3Kγ KO *p* = 0.0043). Cumming estimation plot shows mean differences (lower axes) between controls and experimental groups [P2Y12 KO 0.0423 (95%CI 0.00771, 0.0642); PI3Kγ KO −0.0441 (95%CI −0.0867, −0.00742)]. ***C***, Similar to motility, surveillance in the control group was not different from that in either P2Y12 KO or PI3Kγ KO, but the surveillance of PI3Kγ KO microglia was lower than P2Y12 KO microglia (one-way ANOVA, *p* = 0.043; Tukey’s test: control vs P2Y12KO *p* = 0.24; control vs PI3Kγ KO *p* = 0.34; P2Y12 KO vs PI3Kγ KO *p* = 0.034). Mean differences versus controls shown in Cumming estimation plot [P2Y12 KO 0.023 (95%CI 0.00123, 0.0398); PI3Kγ KO −0.0199 (95%CI −0.0471, 0.00586)]. *n* (animals) = 9 control; 5 P2Y12 KO; 5 PI3Kγ KO); ns, non-significant; *p < 0.05, **p < 0.01. Data are presented as mean ± SEM. Scale bar: 20 μm.

In addition to these static measures of microglia at baseline, we also characterized microglial surveillance of cortical layer 2/3 using *in vivo* two-photon imaging through a chronic cranial window in PI3Kγ KO and P2Y12 KO mice bred with Cx3cr1-EGFP mice to fluorescently label microglia [*n* (mice) = 9 control, 5 P2Y12 KO, 5 PI3Kγ KO; Fig. 3; Movie 2). Although P2Y12 does not appear to be involved in baseline microglial dynamics (Haynes et al., 2006; Madry et al., 2018; Sipe et al., 2016), local production of PIP3 has been implicated in pseudopodia formation and dynamics in other cells (Insall, 2010). Thus, dynamics of the pseudopodia-like end tips of microglial processes may be affected by disruptions to PI3Kγ. There were no significant differences in the motility index, a composite measure of extension and retraction of microglial processes over time (Fig. 3*A*), between controls and either P2Y12 KO or PI3Kγ KO microglia (Fig. 3*B*; control 0.478 ± 0.014; P2Y12 KO 0.520 ± 0.003; PI3Kγ KO 0.433 ± 0.017; Table 1g). Similarly, surveillance, a measurement of the total area covered by microglia and their processes over time, also showed no differences between control and either P2Y12 KO or PI3Kγ KO groups (Fig. 3*C*; control 0.661 ± 0.010; P2Y12 KO 0.684 ± 0.003; PI3Kγ KO 0.641 ± 0.012; Table 1h). While PI3Kγ KO mice had statistically significant lower motility and surveillance relative to P2Y12 KO mice, the fact that neither group was different from control mice leads us to conclude that genetic loss of PI3Kγ did not have an effect on microglial dynamics under homeostatic conditions.

Movie 2. Related to Figure 3. *In vivo* time-lapse imaging of cortical layer 2/3 microglia in the Cx3cr1-EGFP/+, P2Y12 KO Cx3cr1-EGFP/+, or PI3Kγ KO Cx3cr1-EGFP/+ mouse under baseline conditions through a chronic cranial window. Scale bar: 20 μm. One frame per 2 min. 10.1523/ENEURO.0311-20.2020.video.2

### PI3Kγ is not a predominant mediator of the response to focal ATP release *ex vivo*

**Figure 4.**
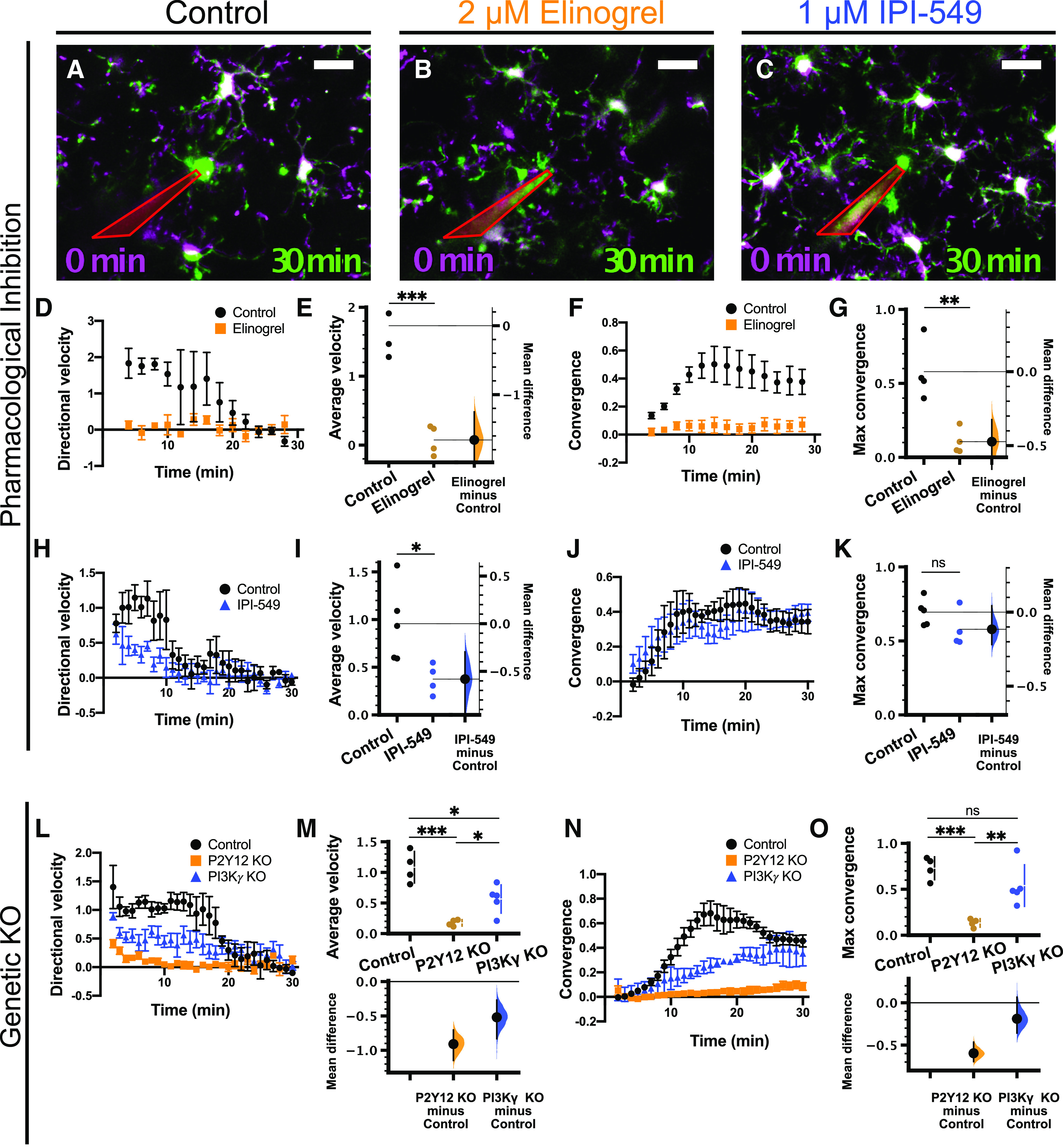
PI3Kγ is not required for the microglial response to focal ATP release in acute cortical slices. Overlays of representative responses from control (***A***), 2 μm elinogrel-treated (P2Y12 inhibitor; ***B***), and 1 μm IPI-549-treated (PI3Kγ inhibitor; ***C***) slices at the time points indicated. Images at later time points (green) are overlaid with initial images (*t* = 0 min, magenta). In ***A–C***, the pipette is outlined in red. ***D–G***, Effects of 2 μm elinogrel on the microglial response to focal ATP release [*n* (slices) = 4 control, 4 elinogrel; from 4 animals total]. ***D***, Time course of directional velocity, with one frame acquired every 2 min. Quantification begins at *t* = 4 min (two frames after puff) to avoid artifacts from the dye included in the pipette solution which leaks into the imaging field at earlier time points. ***E***, Elinogrel completely blocks the directional response [comparisons made on average velocity calculated in the interval of 4–10 min after ATP presentation; unpaired *t* test, *p* = 0.0005; mean difference is −1.66 (95%CI −2.11, −1.25)]. ***F***, Time course of convergence. ***G***, Elinogrel reduces the maximum convergence [unpaired *t* test, *p* = 0.0048; unpaired mean difference is −0.473 (95%CI −0.708, −0.325)]. ***H–K***, Effects of 1 μm IPI-549 on the microglial response to focal ATP release [*n* (slices) = 5 control, 4 IPI-549; from 5 animals total]. ***H***, Time course of directional velocity, with one frame acquired every minute. Quantification begins at *t* = 2 min (two frames after puff). ***I***, IPI-549 partially reduces the directional response [comparisons made on average velocity calculated in the interval of 2–10 min after ATP puff; two-tailed *t* test *p* = 0.031; mean difference is −0.581 (95%CI −0.995, −0.296)]. ***J***, Time course of convergence. ***K***, IPI-549 does not affect the maximum convergence [two-tailed *t* test, *p* = 0.15; mean difference is −0.116 (95%CI −0.219, 0.0431)]. ***L–O***, Effects of P2Y12 KO (orange) or PI3Kγ KO (blue) on microglial response to focal ATP release in slices [*n* (animals) = 4 control; 4 P2Y12 KO; 5 PI3Kγ KO]. ***L***, Time course of directional velocity with one frame acquired every 1 min. ***M***, P2Y12 KO and PI3Kγ KO reduce the directional velocity compared with control [comparisons made on average velocity calculated in the interval of 2–10 min after ATP presentation; one-way ANOVA *p* = 0.0003; Tukey’s test: control vs P2Y12 KO *p* = 0.0002; control vs PI3Kγ KO *p* = 0.0089; P2Y12 KO vs PI3Kγ KO *p* = 0.042; mean differences vs controls: P2Y12 KO −0.909 (95%CI −1.15, −0.706); PI3Kγ KO −0.52 (95%CI −0.832, −0.27)], although the magnitude of the effect is larger in P2Y12 KOs. ***N***, Time course of convergence. ***O***, P2Y12 KO decreases maximum convergence, while PI3Kγ KO does not affect maximum convergence [one-way ANOVA *p* = 0.0011; Tukey’s test: control vs P2Y12 KO *p* = 0.0010; control vs PI3Kγ KO *p* = 0.23; P2Y12 KO vs PI3Kγ KO *p* = 0.0090; mean differences vs controls: P2Y12 KO −0.596 (95%CI −0.693, −0.465); PI3Kγ KO −0.189 (95%CI −0.358, 0.0652)]; ns, non-significant; *p < 0.05, **p < 0.01, ***p < 0.001. Data presented as mean ± SEM. Scale bar: 20 μm. Additional data on the effects of inhibition of actin polymerization or activation of AC on directed microglial motility are presented in Extended Data Figures 4-1, 4-2, respectively.

Next, we wanted to determine whether PI3Kγ is involved in mediating microglial P2Y12-dependent directed motility. Two-photon imaging of microglia in acutely-prepared brain slices is a valuable tool to investigate microglial dynamics and responses to exogenous stimuli. There are two major advantages to the *ex vivo* slice preparation that we took advantage of in this study: (1) the ability to test the direct microglial response to focal ATP release using a glass micropipette, and (2) the ability to pharmacologically manipulate receptors and signaling pathways via bath application of drugs (as exhibited for pan-PI3K inhibition in Fig. 1). Acutely-prepared cortical slices were preincubated with drug or vehicle for ∼15 min. Imaging began immediately before release of focal ATP through the micropipette. Over the course of ∼30 min, microglia responded to focal ATP release by directing processes to the tip of the ATP-containing pipette (Fig. 4*A*; Movie 3). Rhodamine was included in the pipette solution to visualize the expulsion of solution into the parenchyma.

10.1523/ENEURO.0311-20.2020.f4-1 Extended Data Figure 4-1 Microglial chemotactic response in acute cortical slices is dependent on actin polymerization. ***A–D***, Effects of the actin polymerization inhibitor cytochalasin D (1 μM; red squares) on the microglial response to focal ATP release [*n* (slices) = 4 control; 5 cytochalasin D from 5 animals total]. ***A***, Time course of directional velocity. ***B***, Cytochalasin abolishes the directional response [comparisons made on average velocity calculated in the interval of 2–10 min after ATP presentation; Welch’s *t* test, *p* = 0.034; mean difference –0.693 (95%CI –0.994, –0.323)]. ***C***, Time course of microglial process convergence. ***D***, Cytochalasin D reduces the maximum convergence [two-tailed *t* test, *p* = 0.0002; mean difference –0.507 [95%CI –0.629, –0.368)]. ***E–H***, Effects of cytochalasin D (1 μm; red squares) on microglial response to focal laser injury [*n* (slices) = 3 control; 3 cytochalasin D from 3 animals total]. ***E***, Time course of directional velocity. ***F***, Cytochalasin D decreases the directional velocity [average velocity quantified at the interval of 10–20 min after injury; Welch’s, *p* = 0.053; mean difference –1.05 (95%CI –1.36, –0.54)], although this did not reach statistical significance. ***G***, Time course for convergence. ***H***, Convergence at 30 min is also diminished in the presence of cytochalasin D [Welch’s *t* test, *p* = 0.039; mean difference –0.58 (95%CI –0.797, –0.42)]; *p < 0.05, **p < 0.01, ***p < 0.001. Data presented as mean ± SEM. Download Figure 4-1, EPS file.

10.1523/ENEURO.0311-20.2020.f4-2 Extended Data Figure 4-2 Microglial response to focal ATP release in acute cortical slices is not affected by activation of AC. Effects of the AC activator forskolin (10 μm; light green squares) on the microglial response [*n* (slices) = 4 control, 5 forskolin from 6 animals]. ***A***, Time course of directional velocity. ***B***, Forskolin does not significantly affect the directional response [comparisons made on average velocity calculated in the interval of 2–10 min after ATP presentation; unpaired *t* test *p* = 0.069; mean difference –0.325 (95%CI –0.656, –0.0945)]. ***C***, Time course for convergence. ***D***, Forskolin does not significantly affect maximum convergence [unpaired *t* test, *p* = 0.90; mean difference 0.0294 (95%CI –0.415, 0.327)]. Download Figure 4-2, EPS file.

Movie 3. Related to Figure 4. Effect of pharmacological inhibition of P2Y12 with 2 μm elinogrel on microglial response to focal ATP release in an acute cortical slice. Scale bar: 20 μm. One frame per 2 min. 10.1523/ENEURO.0311-20.2020.video.3

As a positive control for our pharmacological experiments, we used the P2Y12 inhibitor elinogrel (2 μm; Oestreich, 2010). Elinogrel treatment completely blocked the response to focal ATP release [*n* (slices) = 4 control, four elinogrel-treated; Fig. 4*B*,*D–F*; Movie 3], as demonstrated by negligible directional velocity (control 1.73 ± 0.22 a.u.; elinogrel 0.07 ± 0.11 a.u.; Fig. 4*E*; Table 1i) and convergence (control 0.58 ± 0.10; elinogrel 0.11 ± 0.04; Fig. 4*J*; Table 1i). This was expected since directed motility to ATP has been shown to be entirely dependent on P2Y12 (Haynes et al., 2006). In addition, the actin polymerization inhibitor cytochalasin D (1 μm) also completely blocked the response to ATP (Extended Data Fig. 4–2; Movie 10; Table 1y), confirming that this process is actin dependent. To inhibit PI3Kγ in slices, we used IPI-549 (1 μm) because of its high potency and high selectivity (>100-fold) over other isoforms of PI3K (Evans et al., 2016; Kaneda et al., 2016). Pharmacological inhibition of PI3Kγ had a modest effect on directional velocity [Fig. 4*H*,*I*; Movie 4; *n* (slices) = 5 control, four IPI-549 treated; average velocity: control 0.96 ± 0.19 a.u.; IPI-549 0.38 ± 0.08; Table 1j], but not convergence (Fig. 4*J*,*K*; max convergence: control 0.69 ± 0.04; IPI-549 0.58 ± 0.06; Table 1j), suggesting slightly impaired process recruitment. In parallel, we conducted similar experiments using P2Y12 KO; Cx3cr1-EGFP/+ or PI3Kγ KO; Cx3cr1-EGFP/+ mice and compared them to Cx3cr1-EGFP/+ animals. Consistent with our previous findings, the response of P2Y12 KO microglia to ATP was almost completely absent [Fig. 4*L-O*, orange; Movie 5; *n* (animals) = 4 control, 4 P2Y12 KO, 5 PI3Kγ KO]. PI3Kγ KO microglia had modestly decreased directional velocity (control 1.08 ± 0.13 a.u.; P2Y12 KO 0.17 ± 0.03 a.u.; PI3Kγ KO 0.56 ± 0.10 a.u.; Fig. 4*M*; Table 1k), while the maximum convergence was not affected (Fig. 4*L–O*, blue; Movie 5; control 0.73 ± 0.06; P2Y12 KO 0.13 ± 0.02; PI3Kγ KO 0.54 ± 0.10; Fig. 4*N*; Table 1l). Our results from both pharmacological and genetic manipulations show a subtle decrease in directional velocity when PI3Kγ is inhibited, but the overall chemotactic response to ATP is still intact. This is in line with findings in peripheral immune cells showing that PI3Kγ is not absolutely required for certain forms of chemotaxis, but may help to fine tune and amplify chemotactic processes (Afonso and Parent, 2011).

Movie 4. Related to Figure 4. Effect of pharmacological inhibition of PI3Kγ with 1 μM IPI-549 on microglial response to focal ATP release in an acute cortical slice. Scale bar: 20 μm. One frame per 1 min. 10.1523/ENEURO.0311-20.2020.video.4

Movie 5. Related to Figure 4. Effects of genetic KO of P2Y12 or PI3Kγ on microglia response to focal ATP release in an acute cortical slice. Scale bar: 20 μm. One frame per 1 min. 10.1523/ENEURO.0311-20.2020.video.5

In addition to its activity as a lipid kinase, PI3Kγ can also act as an A kinase anchoring protein (AKAP), forming a macromolecular complex with PKA and phosphodiesterase 3 (PDE3) to suppress cAMP signaling (Perino et al., 2011; Schmidt et al., 2013). Increasing microglial cAMP, either through inhibition of PDE3B (Bernier et al., 2019) or stimulation of Gs-coupled β2-adrenergic receptors (β2-ARs; Liu et al., 2019; Stowell et al., 2019), leads to retraction of microglial processes. Thus, the observed effects of PI3Kγ blockade on directed microglial motility might be because of increased cAMP. To test this hypothesis, we used a pharmacological activator of adenylate cyclase (AC), forskolin (10 μm; Swiatkowski et al., 2016) to globally increase cAMP in microglia. We found that forskolin had no significant effect on either directional velocity (control 0.73 ± 0.14; forsklin 0.41 ± 0.08; Extended Data Fig. 4-2*A*,*B*; Table 1zz) or convergence (control 0.52 ± 0.13; forskolin 0.55 ± 0.16; Extended Data Fig. 4-2*C*,*D*; Movie 11; Table 1zz) of microglial processes to focal ATP release. These results suggest that cAMP signaling does not play a major role in regulating ATP-mediated directed motility and the effects of inhibition or deletion of PI3Kγ are related to its kinase activity.

### PI3Kγ is not a predominant mediator of the focal damage response

**Figure 5.**
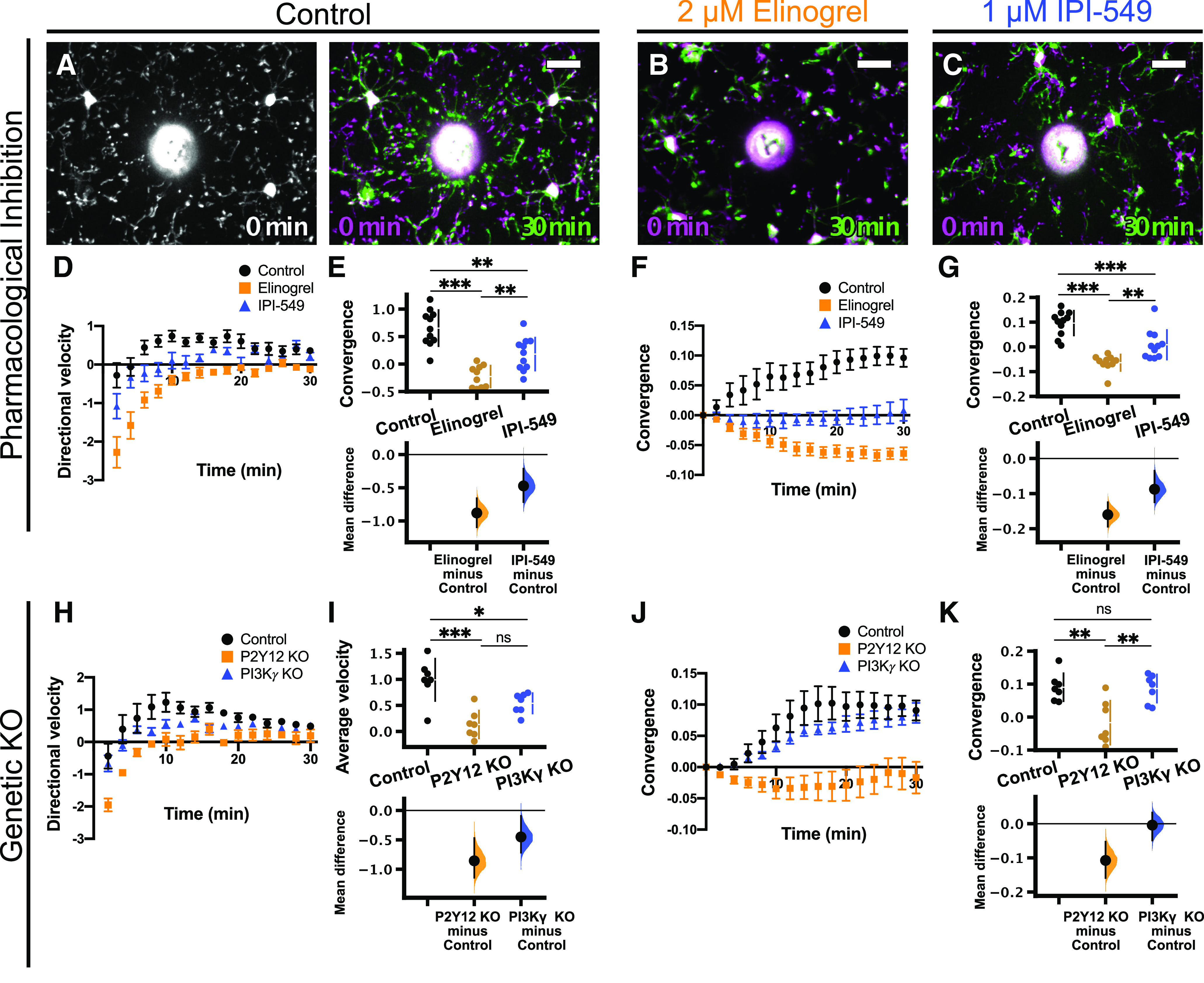
PI3Kγ is not required for the microglial response to laser injury in acute cortical slices. Representative images of the microglial response to laser injury in control (***A***), 2 μm elinogrel-treated (P2Y12 inhibitor; ***B***), and 1 μm IPI-549-treated (PI3Kγ inhibitor; ***C***) slices at the time points indicated. Images at 30 min after injury (green) are overlaid with initial images (*t* = 0 min, magenta). ***D–G***, Effects of 2 μm elinogrel (orange squares) or 1 μm IPI-549 (blue triangles) on the microglial response to laser injury [*n* (slices) = 11 control, 10 elinogrel, 11 IPI-549 from 11 animals total]. ***D***, Time course of directional velocity, one frame every 2 min. Note that the negative directional velocity that occurs soon after ablation is an artifact of tissue deformation following the laser injury. ***E***, IPI-549 partially inhibits the directional response, whereas elinogrel completely blocks it [average velocity quantified at the interval of 10–20 min after injury; one-way ANOVA *p* < 0.0001; Tukey’s test: control vs elinogrel *p* < 0.0001; control vs IPI-549 *p* = 0.0014; elinogrel vs IPI-549 *p* = 0.0071; mean differences vs controls: elinogrel −0.88 (95%CI −1.1, −0.659); IPI-549 −0.472 (95%CI −0.715, −0.214)]. ***F***, Time course for convergence. ***G***, IPI-549 has an intermediate effect on convergence at 30 min, while elinogrel completely blocks convergence [one-way ANOVA *p* < 0.0001; Tukey’s test: control vs elinogrel *p* < 0.0001; control vs IPI-549 *p* = 0.0007; elinogrel vs IPI-549 *p* = 0.0059; mean differences vs controls: elinogrel −0.16 (95%CI −0.194, −0.125); IPI-549 −0.0876 (95%CI −0.125, −0.035)]. ***H–K***, Effects of P2Y12 KO (orange) or PI3Kγ KO (blue) on microglial response to focal injury [*n* (animals) = 7 control, 7 P2Y12 KO, 7 PI3Kγ KO]. ***H***, Time course for directional velocity. ***I***, P2Y12 KO and PI3Kγ KO reduce the directional velocity compared with control [comparison based on average velocity in the interval of 10–20 min after injury; one-way ANOVA *p* = 0.0002; Tukey’s test: control vs P2Y12 KO *p* = 0.0001; control vs PI3Kγ KO *p* = 0.031; P2Y12 KO vs PI3Kγ KO *p* = 0.054; mean differences vs controls: P2Y12 KO −0.856 (95%CI −1.14, −0.47); PI3Kγ KO −0.45 (95%CI −0.717, −0.0926)]. ***J***, Time course for convergence. ***K***, P2Y12 KO decreases convergence at 30 min, while PI3Kγ KO has no effect [one-way ANOVA *p* = 0.0014; Tukey’s test: control vs P2Y12 KO *p* = 0.0030; control vs PI3Kγ KO *p* = 0.99; P2Y12 KO vs PI3Kγ KO *p* = 0.0041; mean differences vs control: P2Y12 KO −0.107 (95%CI −0.159, −0.0526); PI3Kγ KO −0.00402 (95%CI −0.0487, 0.0325)]; ns, non-significant; *p < 0.05, **p < 0.01, ***p < 0.001. Data presented as mean ± SEM. Scale bar: 20 μm.

We also analyzed the microglial response to focal laser injury using the directional velocity and convergence analyses. Although microglial responses to focal injury and direct application of ATP are likely influenced by different extracellular cues, both responses involve the rapid movement of microglial processes toward the site of injury in a P2Y12-dependent manner (Haynes et al., 2006). We first repeated our pharmacological and genetic manipulations to disrupt P2Y12 and PI3Kγ function in acute brain slices and observed the effects on microglial directed motility toward the ablation. As expected, the recruitment of microglial processes to the injury core was dependent on actin remodeling, and treatment with cytochalasin D blocked microglial directed motility (Extended Data Fig. 4-2; Movie 10; Table 1z). Inhibition with elinogrel (2 μm) or genetic deletion of P2Y12 also completely abolished the microglial response (Fig. 5, orange; Movies 6, 7), as expected from our experiments with focal ATP application and previous experiments in the literature. The role of PI3Kγ was less pronounced. PI3Kγ inhibition with IPI-549 (1 μm; Fig. 5, blue; Movies 6, 7) had an intermediate effect on both directional velocity (control 0.65 ± 0.10 a.u.; elinogrel −0.22 ± 0.06 a.u.; IPI-549 treatment 0.18 ± 0.09 a.u.; Table 1m) and convergence (control 0.096 ± 0.015; elinogrel −0.063 ± 0.010; IPI-549 0.009 ± 0.018; Table 1n), while PI3Kγ KO microglia showed a slightly decreased directional velocity (control 0.99 ± 0.15 a.u.; P2Y12 KO 0.13 ± 0.10 a.u.; PI3Kγ KO 0.54 ± 0.36 a.u.; Table 1o) without an effect on convergence (control 0.091 ± 0.015; P2Y12 KO −0.017 ± 0.025; PI3Kγ KO 0.086 ± 0.016; Table 1p).

Movie 6. Related to Figure 5. Effects of pharmacological inhibition of P2Y12 (with 2 μm elinogrel) or PI3Kγ (with 1 μM IPI-549) on the microglial response to focal laser injury in an acute cortical slice. Scale bar: 20 μm. One frame per 2 min. 10.1523/ENEURO.0311-20.2020.video.6

Movie 7. Related to Figure 5. Effects of genetic KO of P2Y12 or PI3Kγ on the microglial response to focal laser injury in an acute cortical slice. Scale bar: 20 μm. One frame per 2 min. 10.1523/ENEURO.0311-20.2020.video.7

**Figure 6.**
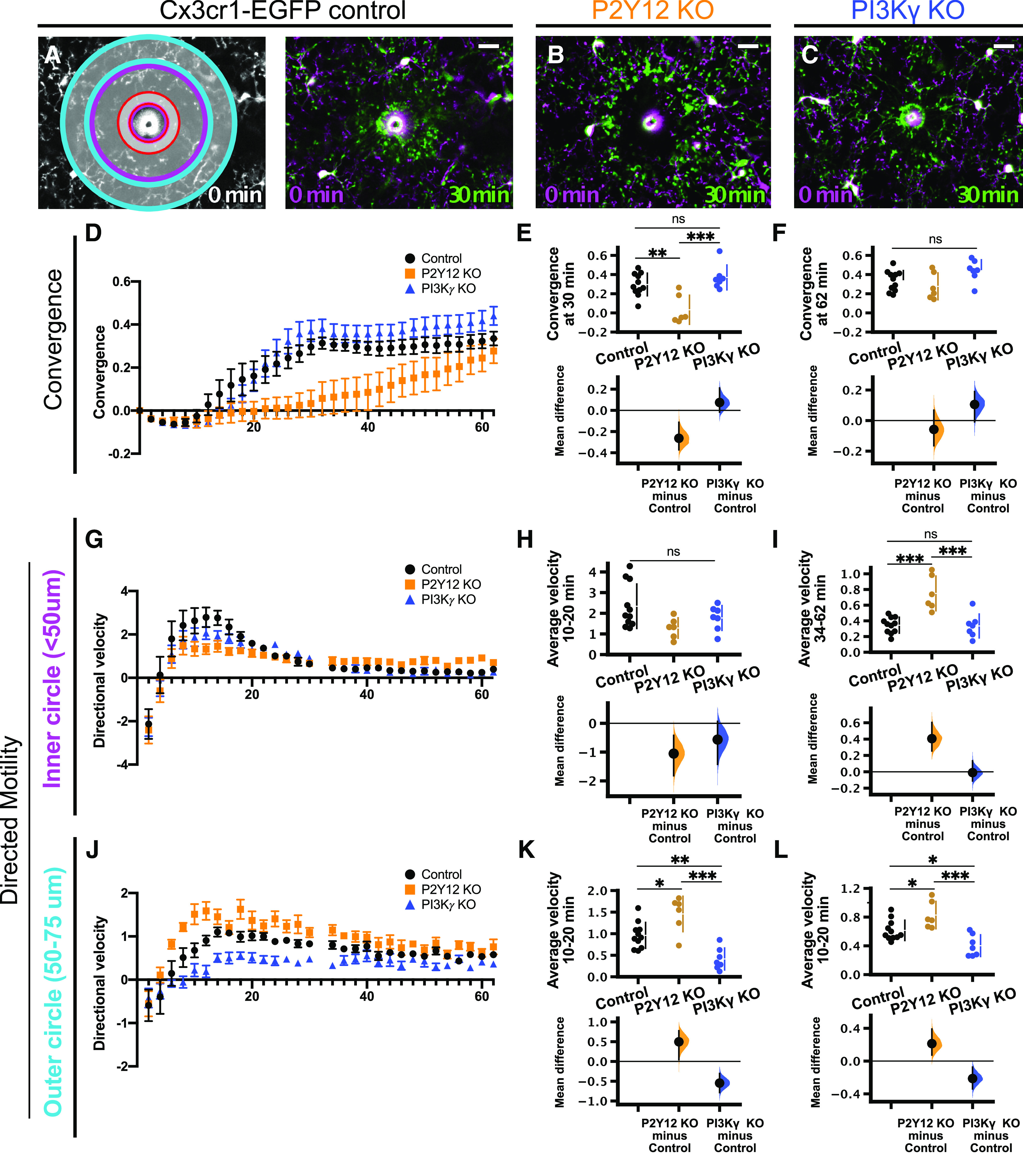
PI3Kγ is not required for the microglial response to laser injury *in vivo*. Representative images of the microglial response to laser injury *in vivo* in control (***A***, *n* = 11 mice), P2Y12 KO (***B***, *n* = 6 mice), and PI3Kγ KO (***C***, *n* = 7 mice) at the time points indicated. Images at 30 min after injury (green) are overlaid with initial images (*t* = 0 min, magenta). The first panel shows the approximate regions of interest used for each type of analysis: convergence (red), inner circle motility (purple), and outer circle motility (cyan). ***D***, Time course of convergence over a 62-min imaging session. ***E***, Convergence at 30 min is similar in control and PI3Kγ KO groups, while P2Y12 KO microglia do not converge at this time point [one-way ANOVA *p* = 0.0003; Tukey’s test: control vs P2Y12 KO *p* = 0.0018; control vs PI3Kγ KO *p* = 0.48; P2Y12 KO vs PI3Kγ KO *p* = 0.0004; mean differences vs control: P2Y12 KO −0.263 (95%CI −0.372, −0.112); PI3Kγ KO 0.0741 (95%CI −0.0165, 0.211)]. ***F***, By 62 min, convergence across all groups is similar [one-way ANOVA, *p* = 0.055; mean differences vs control: P2Y12 KO −0.058 (95%CI −0.165, 0.068); PI3Kγ KO 0.105 (95%CI −0.00849, 0.19)]. ***G***, Time course of directional velocity within the inner circle (<50 μm from center of injury). Note there is a missing time point at *t* = 32 min because of a discontinuity in image acquisition that affects the optic flow calculations. ***H***, There were no differences between genotypes in the directional velocity during the peak of the response [average velocity calculated in the interval of 10–20 min after injury; one-way ANOVA *p* = 0.063; mean differences vs control: P2Y12 KO −0.058 (95%CI −0.165, 0.068); PI3Kγ KO 0.105 (95%CI −0.00849, 0.19)]. ***I***, However, P2Y12 KO microglia had sustained movement toward the injury site, which was significantly higher than control or PI3Kγ KO groups [average velocity calculated in the interval of 34–62 min; one-way ANOVA *p* < 0.0001; Tukey’s test: control vs P2Y12 KO *p* < 0.0001; control vs PI3Kγ KO *p* = 0.99; P2Y12 KO vs PI3Kγ KO *p* = 0.0002; mean differences vs control: P2Y12 KO 0.406 (95%CI 0.255, 0.603); PI3Kγ KO −0.00985 (95%CI −0.112, 0.134)]. ***J***, Time course of directional velocity within the outer circle (50–75 μm from injury center). ***K***, P2Y12 KO mice showed increased directional velocity during the peak of the response (10–20 min) compared with control, while PI3Kγ KO show decreased directional velocity during this time [one-way ANOVA *p* < 0.0001; Tukey’s test: control vs P2Y12 KO *p* = 0.0152; control vs PI3Kγ KO *p* = 0.0051; P2Y12 KO vs PI3Kγ KO *p* < 0.0001; mean differences vs control: P2Y12 KO 0.497 (95%CI 0.0439, 0.776); PI3Kγ KO −0.548 (95%CI −0.787, −0.305)]. ***L***, These differences in directional response were sustained later in the injury response [34–62 min; one-way ANOVA *p* = 0.0003; Tukey’s test: control vs P2Y12 KO *p* = 0.036; control vs PI3Kγ KO *p* = 0.027; P2Y12 KO vs PI3Kγ KO *p* = 0.0002; mean differences vs control: P2Y12 KO 0.213 (95%CI 0.0734, 0.391); PI3Kγ KO −0.213 (95%CI −0.339, −0.0739)]. Comparisons (***E***, ***F***, ***H***, ***I***, ***K***, ***L***) done using one-way ANOVA with Tukey’s *post hoc* comparisons; ns, non-significant; *p < 0.05, **p < 0.01, ***p < 0.001. Data presented as mean ± SEM. Scale bar: 20 μm. Heat maps of directional velocity as a function of both time and distance from injury are presented in Extended Data Figure 6-1.

We then examined the microglial response to focal injury *in vivo*, imaging layer 2/3 cortical microglia through a chronic cranial window. Interestingly, microglial responses to injury were slower and more sustained *in vivo* when compared with those in acute slices (Figs. 5 vs 6) suggesting either changes in microglial function after acute slice preparation, or in chemotactic gradients and injury. We therefore monitored the microglial response for 1 h after injury was induced, to fully capture the chemotactic response [*n* (animals) = 11 control, 6 P2Y12 KO, 7 PI3Kγ KO]. Consistent with the results of our previous *ex vivo* experiments, rapid convergence (at 30 min) was impaired in the P2Y12 KO animals, but not in the PI3Kγ KO animals (Fig. 6*D*,*E*; Movie 8; control 0.296 ± 0.036; P2Y12 KO 0.033 ± 0.062; PI3Kγ KO 0.370 ± 0.049; Table 1q). By the end of the imaging session, the P2Y12 KO microglia did converge around the injury site (Fig. 6*F*; convergence at 62 min: control 0.335 ± 0.032; P2Y12 KO 0.277 ± 0.057; PI3Kγ KO 0.441 ± 0.043; Table 1r), consistent with previous findings that the P2Y12-independent response is much slower than the response mediated by P2Y12 (Haynes et al., 2006). The *in vivo* response to focal injury was more robust and complex than in acute brain slices, as evidenced by a much larger magnitude of convergence, possibly because of the absence of confounding injury signals from the slicing procedure and restricted diffusion in the intact brain. We therefore more closely analyzed the relationship between the directional velocity, time, and distance from the injury site to tease out the involvement of P2Y12 and PI3Kγ in different aspects of the response (Extended Data Fig. 6-1). In control animals, there was an initial wave of coordinated process movement, in which processes were recruited from an area relatively proximal to the injury (within ∼45 μm) and converged on the ablation core in ∼20–30 min. However, there was also considerable process movement farther from the injury site (>50 μm) that had a different dynamic profile, was sustained throughout the imaging session and persisted beyond the time when convergence had plateaued. Thus, in our comparisons across genotypes, we separated out the directional velocity by distance from the center to capture these complex dynamics (magenta vs cyan; Fig. 6*G–L*). Surprisingly, we found a high directional velocity in the P2Y12 KO animals (average velocity <50 μm from injury site: 10–20 min: control 02.34 ± 0.32 a.u.; P2Y12 KO 1.29 ± 0.20 a.u.; PI3Kγ KO 1.77 ± 0.23 a.u; Table 1s; 34–62 min: control 0.34 ± 0.03 a.u.; P2Y12 KO 0.75 ± 0.09 a.u.; PI3Kγ KO 0.33 ± 0.06 a.u.; Table 1t). P2Y12 KO microglial processes oriented and moved toward the focal injury especially during the early response, but their convergence was impaired. Likely because of this impaired convergence, the long-range directional velocity in P2Y12 KO microglia was higher than control or PI3Kγ KO groups (Fig. 6*J–L*; average velocity 50–75 μm from injury site: 10–20 min: control 0.96 ± 0.09 a.u.; P2Y12 KO 1.46 ± 0.17 a.u.; PI3Kγ KO 0.41 ± 0.10 a.u.; Table 1u; 34–62 min: control 0.62 ± 0.04 a.u.; P2Y12 KO 0.83 ± 0.08 a.u.; PI3Kγ KO 0.40 ± 0.06 a.u.; Table 1v), as the initially responsive microglial processes remained farther away from the center. These differences in the spatiotemporal dynamics of the response in the P2Y12 KO mice can be appreciated by examining the plot of directional velocity as a function of both time and distance from the injury site (Extended Data Fig. 6–1*B*). Accompanying these dynamics, there was a change in the appearance of the microglial process end tips, which become broader as they approach the injury site. This underscores the fact that P2Y12 KO microglia were still able to react to the tissue damage, although in a substantially impaired manner. For the PI3Kγ KO animals, the directional velocity within 50 μm was not different from control animals (Fig. 6*G-I*). However, the recruitment of more distant processes appeared to be impaired, as demonstrated by a decreased directional velocity >50 μm from the injury that was persistent over the course of 60 min (Fig. 6*J-L*). Our *in vivo* data suggest that while the overall response to focal injury is not impaired in PI3Kγ KO mice, there are subtle defects in long-range process recruitment, where the chemotactic signals are likely weaker than in proximal areas.

10.1523/ENEURO.0311-20.2020.f6-1 Extended Data Figure 6-1 Directional velocity as a function of time and distance from the injury core *in vivo*. These graphs depict the same experiments as are presented in Figure 6. To visualize the spatiotemporal dynamics of the *in vivo* laser injury response, the average directional velocity was calculated as a function of both time and distance from the center of the core (“radius”). Concentric circles were generated with radii increasing in intervals of 5 μm. The directional velocities between two adjacent circles (e.g., between 50 to 55 μm) were averaged, generating 5-μm sized bins, where a radius of 55 μm would denote the velocities between 50 and 55 μm. These calculations were done for each sample and then averaged together for each genotype: control (***A***, *n* = 11 mice), P2Y12 KO (***B***, *n* = 6 mice), and PI3Kγ KO (***C***, *n* = 7 mice). The heatmaps were then generated in MATLAB, and the plots smoothed by interpolating between points. All graphs are plotted with the same color map scale. This analysis shows that the P2Y12 KO group (***B***) has a fundamentally different response than the other two genotypes, with most movement restricted to an area far (>40 μm) from the injury. The PI3Kγ KO group (***C***) has a similar response to the controls, especially closer to the injury with reduced directed motility farther from the ablation core when compared to the controls (also see Figure 6*J–L*). Download Figure 6-1, EPS file.

Movie 8. Related to Figure 6. Effects of genetic KO of P2Y12 or PI3Kγ on the microglial response to focal laser injury in cortical layer 2/3 microglia *in vivo*. Scale bar: 20 μm. One frame per 2 min. 10.1523/ENEURO.0311-20.2020.video.8

Movie 9. Related to Extended Data Figure 1-1. Color coded velocity vectors (green = net towards center; red = net away from center) overlaid on top of a video of the microglial response to focal injury *in vivo*. Scale bar: 20 μm. One frame per 2 min. 10.1523/ENEURO.0311-20.2020.video.9

Movie 10. Related to Extended Data Figure 4-1. Effects of inhibition of actin polymerization (with 1 μm cytochalasin D) on the response of microglia to focal ATP release and focal laser injury in an acute cortical slice. Scale bar: 20 μm. One frame per 1 min. 10.1523/ENEURO.0311-20.2020.video.10

Movie 11. Related to Extended Data Figure 4-2. Effects of activation of AC (with 10 μm forskolin) on the response of microglia to focal ATP release in an acute cortical slice. Scale bar: 20 μm. One frame per 1 min. 10.1523/ENEURO.0311-20.2020.video.11

Overall, inhibition or absence of PI3Kγ caused subtle defects in microglial process recruitment toward sources of ATP. Given the effects of pharmacological inhibition or genetic KO experiments were similar across multiple experimental paradigms, PI3Kγ likely plays a minor role in ATP-mediated directed microglial motility.

### PI3Kγ is not required for ocular dominance plasticity

**Figure 7.**
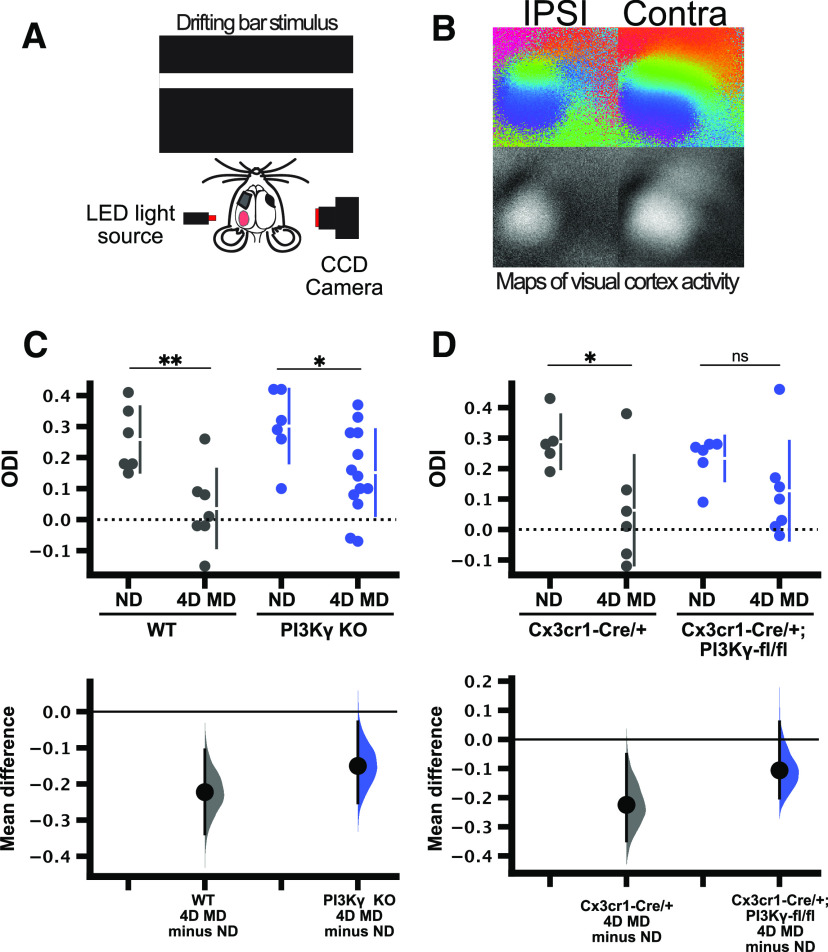
PI3Kγ is not required for ocular dominance plasticity. ***A***, Schematic of IOS imaging rig (see Materials and Methods). ***B***, Representative phase (top) and amplitude (bottom) maps of V1 in response to visual stimulus for the ipsilateral (left) and contralateral (right) eyes. Phase maps demonstrate retinotopic organization, while amplitude maps show normalized hemodynamic response to the stimulus. ***C***, Effects of global PI3Kγ KO on ocular dominance plasticity [*n* (animals) = 6 WT ND; 7 WT 4D MD; 6 PI3Kγ KO ND; 13 PI3Kγ KO 4D MD]. WT (gray) and PI3Kγ KO (blue) mice were either ND or underwent 4D MD, and the ODI for each mouse was calculated as ODI = (contra response – ipsi response)/(contra response + ipsi response). Both the WT and the PI3Kγ KO showed significant shifts in ODI following 4D MD [two-way ANOVA; main effect of deprivation *p* = 0.0005; Sidak’s test: WT ND vs 4D MD *p* = 0.0079; PI3Kγ KO ND vs 4D MD *p* = 0.0472; main effect of genotype *p* = 0.10; mean differences: WT ND vs 4D MD −0.223 (95%CI −0.338, −0.106); PI3Kγ KO ND vs 4D MD −0.15 (95%CI −0.252, −0.0282)]. ***D***, Effects of microglial specific PI3Kγ KO on ocular dominance plasticity. Tamoxifen-treated Cx3cr1-Cre/+ control mice (control, gray) or Cx3cr1-Cre/+ PI3Kγ fl/fl mice (PI3Kγ cKO, blue) underwent ND or 4D MD [*n* (animals) = 5 Cx3cr1-Cre/+ ND; 6 Cx3cr1-Cre/+ 4D MD; 6 Cx3cr1-Cre/+ PI3Kγ fl/fl ND; 7 Cx3cr1-Cre/+ PI3Kγ fl/fl 4D MD]. Control mice showed a significant shift in ODI after 4D MD, while microglia-specific PI3Kγ KO mice showed a trend toward a decrease in ODI [two-way ANOVA: main effect of deprivation *p* = 0.0084, Sidak’s test: Cx3cr1-Cre/+ ND vs 4D MD *p* = 0.0275; Cx3cr1-Cre/+ PI3Kγ fl/fl ND vs 4D MD *p* > 0.05; main effect of genotype *p* = 0.94; mean differences Cx3cr1-Cre/+ ND vs 4D MD −0.225 (95%CI −0.348, −0.051); Cx3cr1-Cre/+ PI3Kγ fl/fl −0.106 (95%CI −0.201, 0.0602)]; ns, non-significant; *p < 0.05, **p < 0.01. Data presented as mean ± SEM. Confirmation of PI3Kγ fl/fl excision and representative amplitude maps for each sample are shown in Extended Data Figures 7-1, 7-2, respectively.

We observed a subtle defect in process recruitment when PI3Kγ was inhibited or genetically deleted. However, these experiments involve either exogenous ATP or pathologic injury and thus likely involve high levels of P2Y12 signaling. Thus, we sought to determine whether PI3Kγ is required for critical-period ocular dominance plasticity, a P2Y12-dependent process (Sipe et al., 2016) that is widely used as a model of physiological experience-dependent synaptic plasticity with neuronal mechanisms resembling long-term potentiation and depression (Cooke and Bear, 2014). The mouse binocular primary visual cortex (V1b) receives stronger inputs from the contralateral eye than the ipsilateral eye, thus giving it a positive ODI (see Materials and Methods). However, during a critical period in adolescence (∼P25–P35), 4D MD of the contralateral eye causes a shift in the ODI toward zero because of depression of the contralateral response (Gordon and Stryker, 1996; Kalatsky and Stryker, 2003). This shift is absent in P2Y12 KO animals and when P2Y12 signaling is inhibited pharmacologically (Sipe et al., 2016). To determine whether loss of PI3Kγ also disrupted ocular dominance plasticity, we monocularly deprived PI3Kγ KO mice for 4 d and used IOS imaging to monitor the cortical responses to visual stimulation of each eye independently (Fig. 7). We found that PI3Kγ KO mice demonstrated a significant shift in ODI after 4D MD [Fig. 7*C*; Extended Data Figs. 7-1, 7–2; genotype (*n* animals) ODI: WT ND (*n* = 6) 0.26 ± 0.04; WT 4DMD (*n* = 7) 0.04 ± 0.05; PI3Kγ KO ND (*n* = 6) 0.30 ± 0.05; PI3Kγ KO 4DMD (*n* = 13) 0.15 ± 0.05; Table 1w], although the magnitude of the shift appeared to be qualitatively smaller than that in control C57Bl/6 mice.

10.1523/ENEURO.0311-20.2020.f7-1 Extended Data Figure 7-1 Confirmation of floxed PI3Kγ excision in microglia in tamoxifen-treated CX3CR1-cre/PI3Kγ fl/fl mice. ***A–E***, Example of microglia FACS. ***A–C***, Size and doublet exclusion. ***D***, Selection for live cells (PI-negative). ***E***, Selection of CD11b-hi; CD45-lo cells. ***F***, Ethidium bromide gel with PCR products of DNA from sorted microglia. Two separate reactions were performed on each sample. Left, PCR for the WT and floxed PI3Kγ (PI3Kγ fl/fl) alleles. Right, PCR for the excision product following Cre-mediated excision of floxed PI3Kγ. Samples include (from left to right): no-template control (NTC); Cx3cr1-Cre/+ PI3Kγ-WT to demonstrate detection of WT allele, Cx3cr1-WT PI3Kγ-fl/fl to demonstrate detection of floxed PI3Kγ allele; two separate animals of Cx3cr1-Cre/+; PI3Kγ-fl/fl (i.e., conditional KOs) treated with tamoxifen as described in Materials and Methods. The absence of PI3Kγ fl or WT allele and the presence of the excision product in the conditional KOs confirms PI3Kγ excision in microglia. Excision was confirmed in this manner from mice from three different litters. Download Figure 7-1, EPS file.

While PI3Kγ expression is largely restricted to microglia in the CNS as assessed by RNAseq of acutely isolated cells (Zhang et al., 2014), there is evidence that it is also expressed in subsets of neurons (Kim et al., 2011; D'Andrea et al., 2015). ODP is a complex process involving the coordinated activity of inhibitory and excitatory neurons (Kuhlman et al., 2013) and is influenced by neuromodulators (Stowell et al., 2019). Thus, global KO of PI3Kγ could have unintended effects in the context of ODP. In order to preserve any non-microglial PI3Kγ signaling and prevent any compensation mechanisms that may be activated in early development because of a global KO, we crossed the PI3Kγ floxed mouse (PI3Kγ-fl/fl) with the Cx3cr1-CreERT2 mouse to generate a microglial-specific PI3Kγ KO mouse line (Cx3cr1-Cre/+; PI3Kγ fl/fl). Cre-mediated excision of PI3Kγ was initiated by three daily doses of tamoxifen delivered via oral gavage on P2–P4. We subjected these microglia-specific PI3Kγ KO mice to 4D MD and measured the cortical responses to visual stimuli with IOS imaging (Fig. 7*D*; Extended Data Fig. 7-2*C*,*D*). We found that tamoxifen-treated Cx3cr1-Cre/+ control mice showed a significant shift in the ODI following 4D MD. In tamoxifen-treated Cx3cr1-Cre/+; PI3Kγ fl/fl mice, 4D MD decreased the ODI compared with the ND group, but this was not statistically significant after correction for multiple comparisons [genotype (*n* animals) ODI: Cx3cr1-Cre/+ ND (*n* = 5) 0.29 ± 0.04; Cx3cr1-Cre/+ 4DMD (*n* = 6) 0.06 ± 0.07; Cx3cr1-Cre/+ PI3Kγ fl/fl ND (*n* = 6) 0.23 ± 0.03; Cx3cr1-Cre/+ PI3Kγ fl/fl 4DMD (*n* = 7) 0.13 ± 0.06; Table 1x]. This suggests that MD-induced ODI shifts may still occur in these mice but may be less robust than when PI3Kγ is present in microglia.

10.1523/ENEURO.0311-20.2020.f7-2 Extended Data Figure 7-2 Representative amplitude maps from IOS imaging experiments. Magnitude maps of BOLD response to a periodic stimulus, related to the data presented in Figure 7. For each genotype, ipsilateral (top) and contralateral (bottom) amplitude example maps are shown for a ND (left) and 4D MD (right) animals. ***A***, ***B***, Corresponds to Figure 7*C*, examining ODP in global PI3Kγ KO mice. ***C***, ***D***, Corresponds to Figure 7*D*, examining ODP in microglial-specific PI3Kγ KO mice. Download Figure 7-2, EPS file.

Thus, the results from both the global and microglia-specific PI3Kγ KO are similar: in the absence of PI3Kγ, ODI shifts still occurred following monocular deprivation, but were not as prominent as in control animals. We conclude that PI3Kγ is not required for ocular dominance plasticity, although its activity may regulate some aspects of plasticity and thus its absence may have subtle effects.

## Discussion

Many recent studies have shown that the G_i_-coupled receptor P2Y12 mediates microglia-neuron interactions in both physiological and pathologic contexts (Eyo et al., 2014; Sipe et al., 2016; Fekete et al., 2018; Zhang and Li, 2019; Cserép et al., 2020). P2Y12 mediates the microglial chemotactic response to exogenous ATP *in vitro*, *ex vivo*, and *in vivo* (Haynes et al., 2006), suggesting that ATP may be released by neurons or glia to attract microglial processes via P2Y12 activation. Despite the breadth of research on the many functional roles of P2Y12 in the CNS, relatively little is known about the intracellular signaling that mediates these P2Y12-dependent processes in microglial cells when they perform their regular functions. In this study, we sought to interrogate the role of PI3Kγ as a putative downstream effector of P2Y12 in both ATP-mediated microglial directed motility and experience-dependent synaptic plasticity. Understanding the mechanisms underlying P2Y12 signaling in microglia can provide insight into how extracellular nucleotides are used in the CNS to facilitate communication and interactions between diverse cell types.

### PI3Kγ in microglia

We investigated PI3Kγ as a possible downstream mediator of P2Y12 signaling because of its activation by Gβγ, which drives many chemotactic pathways downstream of G_i_ activation (Lin and Smrcka, 2011). Thus, PI3Kγ could serve as a link between G-protein activation and a variety of downstream regulators of chemotaxis and directed motility. PI3Kγ has been shown to be an important mediator of functions that are critical to microglial roles in the CNS in both physiological and pathologic settings such as motility, phagocytosis, and microglial responses to pathologic insults. *In vitro*, PI3Kγ-mediated phosphorylation of PIP2 to generate PIP3 in the plasma membrane is required for C5a-induced microglial motility (Schneble et al., 2017). C5a is a potent chemoattractant for microglia that acts via the G_i_-coupled C5a receptor, suggesting PI3Kγ is activated downstream of some G_i_-coupled proteins in microglia (Schneble et al., 2017). Also *in vitro*, deletion of PI3Kγ impairs phagocytosis (Schmidt et al., 2013). Furthermore, inhibition of PI3Kγ can ameliorate some of the proinflammatory and neurotoxic effects of amyloid-β peptides *in vivo* (Passos et al., 2010), suggesting PI3Kγ may play a role in neurodegeneration.

While canonical PI3Kγ signaling involves its kinase activity, such as during C5a-induced motility (Schneble et al., 2017), some of the functions ascribed to PI3Kγ in microglia, especially in pathologic contexts, have been linked to its kinase-independent activity as a scaffolding protein, where it acts as a regulator of cAMP signaling. PI3Kγ is an AKAP, forming a macromolecular complex with PKA and PDE3 (Perino et al., 2011). This interaction allows PKA to phosphorylate and activate PDE3, which degrades cAMP, and leads to termination of cAMP signaling, albeit in a highly localized fashion (Perino et al., 2011). In cultured microglia, deletion of PI3Kγ leads to increased cAMP levels and impaired phagocytosis, a phenotype that is rescued by the expression of a knock-in kinase-dead mutant PI3Kγ that has preserved scaffolding function (Schmidt et al., 2013). This kinase-independent function of PI3Kγ seems to restrict pathology in disease models by regulating matrix metalloproteinase expression (Frister et al., 2014; Schmidt et al., 2016). Thus, this kinase-independent scaffolding function of PI3Kγ and its regulation of cAMP may be induced in response to pathologic insults, as has been described in cardiomyocytes (Perino et al., 2011). We found that pharmacological inhibition (with IPI-549) or genetic deletion of PI3Kγ affected ATP-mediated directed microglial motility in a similar fashion (Figs. 4, 5). While the kinase-independent activity of PI3Kγ was not measured during the development of IPI-549, we can speculate that IPI-549 does not have a large effect on kinase-independent activity because it is likely an ATP-competitive inhibitor (Evans et al., 2016). We conclude that PI3Kγ is likely to subtly regulate directed motility through a kinase-dependent mechanism, although effects of IPI-549 on kinase-independent activity cannot be absolutely ruled out.

### Intracellular signaling in P2Y12-dependent chemotaxis and directed motility

The microglial response to exogenous ATP was first characterized *in vitro*, where gradients of ATP induce membrane ruffles and actin polymerization in microglia along with whole-cell migration toward the nucleotide source (Honda et al., 2001). These effects on the cytoskeleton and motility of microglia are dependent on P2Y12 (Haynes et al., 2006). In the context of *in vitro* whole-cell chemotaxis, it seems ATP (via P2Y12) activates PI3K and phospholipase C (PLC), which in turn act synergistically to activate Akt to drive chemotaxis (Ohsawa et al., 2007; Irino et al., 2008). Precisely how activation of these intracellular signals translates to directional cell movement in microglia has not been tested. One possibility is that this pathway leads to the focal activation of integrins, which promote adhesion to the extracellular matrix and are important for certain types of cellular migration (Yamada and Sixt, 2019). In microglial cultures, P2Y12 activation induces integrin-β1 translocation to sites of actin polymerization and integrin-β1 is required for ADP-induced chemotaxis (Nasu-Tada et al., 2005; Ohsawa et al., 2010).

It is unclear how much the pathways delineated *in vitro* contribute to directed motility of microglia in their native brain environment. Relative to their *in vivo* counterparts, microglia in culture have a substantially different transcriptomic profile (Butovsky et al., 2014) and the response to ATP is qualitatively different. Microglia in acute slices or *in vivo* react to ATP through the rapid extension of their processes toward the source (Davalos et al., 2005; Nimmerjahn et al., 2005; Haynes et al., 2006), a phenomenon that is absent in cultured microglia, which show very little process ramification. Despite these differences, we found that this form of directed motility, which is more physiological, also requires PI3K activity (Fig. 1), as has been previously shown (Wu et al., 2007). However, inhibition or deletion of PI3Kγ had only subtle effects on ATP-mediated directed motility in acute slices or *in vivo* (Figs. 4-6), suggesting that direct activation of PI3Kγ by Gβγ is not strictly necessary for directed motility, and that other PI3K isoforms may be activated indirectly by P2Y12 signaling. In peripheral immune cells, there are positive feedback loops between PI3K and actin polymerization, such that PI3K can act as a downstream amplifier of the chemotactic response independent of the initial signaling event (Charest and Firtel, 2006; Yoo et al., 2010). A similar mechanism may help to explain the discrepancy in the effects of pan-PI3K and PI3Kγ-specific inhibition on the ATP-mediated microglial directed motility.

In parallel to PI3K signaling, several alternative signaling modules can also mediate chemotaxis in eukaryotic cells (Artemenko et al., 2014), acting synergistically or independently of PI3K to elicit the directed motility of microglial processes. Inhibition of the mitogen-associated protein kinase (MAPK) Erk, which constitutes one such pathway, did not affect ATP-mediated directed motility in acute slices (Wu et al., 2007). Another candidate may be the p38 MAPK pathway, which was shown to be activated downstream of P2Y12 in spinal cord microglia in models of neuropathic pain (Kobayashi et al., 2008; Tatsumi et al., 2015). The role of p38 MAPK in microglial directed motility remains to be tested. Release of intracellular Ca^2+^ stores downstream of PLC activation may also play a role. *In vitro*, ATP induces intracellular Ca^2+^ signals and inhibition of PLC blocks ATP-mediated chemotaxis. (Irino et al., 2008). *In vivo*, microglial Ca^2+^ signals are induced by ATP or tissue damage (Eichhoff et al., 2011; Pozner et al., 2015). However, the presence of Ca^2+^ signals does not affect the speed of directed microglial motility toward focal injury (Pozner et al., 2015), and whether inhibition of PLC affects directed microglial motility has not been investigated. Separately, negative regulation of cAMP signaling by P2Y12 could also play a role in directed microglial motility, as increasing cAMP is linked to alterations in microglial motility and surveillance (Bernier et al., 2019; Liu et al., 2019; Stowell et al., 2019). However, pharmacological activation of AC to increase cAMP had little effect on directed microglial motility toward focal ATP (Extended Data Fig. 4-2), which suggests Gα_i_-mediated regulation of AC does not play a role in directed motility toward ATP. An additional target of P2Y12 signaling is THIK-1, a K^+^ channel that mediates an ATP-induced outward current (Wu et al., 2007; Madry et al., 2018). While THIK-1 regulates basal microglial morphology and surveillance, it is not required for directed motility toward ATP sources. THIK-1-mediated K^+^ efflux after P2Y12 stimulation amplifies inflammasome activity (Madry et al., 2018), suggesting a motility-independent effect of P2Y12 signaling. Precisely which of the parallel and possibly redundant second-messenger pathways is active during P2Y12-mediated directed motility and their downstream effectors remains to be determined. We found that actin polymerization was essential (Extended Data Fig. 4-1), and others have shown that volume-sensitive chloride channels are required (Hines et al., 2009). More work is needed to define the cellular machinery that mediates ATP-dependent directed motility.

Unlike the microglial response to ATP, the loss of P2Y12 did not abolish the response to focal injury. P2Y12 KO microglia robustly responded to focal injury *in vivo*, although rapid convergence on the injury site was severely impaired (Fig. 6; Extended Data Fig. 6-1; Movie 8). The delayed convergence (1–2 h after injury) has also been previously described (Haynes et al., 2006). However, the degree of directed motility even early in the injury response has not, to our knowledge, been previously reported. One feature of this P2Y12-independent injury response was the ability of microglial processes to orient and move toward the injury until they were about ∼40 μm from the center, at which point the they appeared to pause and only slowly converge on the core toward the end of the imaging session (Movie 8). Another striking feature was the appearance of the process end tips in P2Y12 KO microglia converging on the core which were wider and less bulbous than the tips of control microglia, reminiscent more of lamellipodia versus filopodia (Fig. 6). Interestingly, cells can switch modes of migration depending on the extracellular environment and activation of different intracellular signaling cascades (Yamada and Sixt, 2019). Thus, the absence of P2Y12 signaling during this damage response may lead to a qualitatively different form of motility and likely relies on non-purinergic chemotactic cues, given the absence of any response to focal ATP release in P2Y12 KO mice (Fig. 4; Haynes et al., 2006). A recent study of the *in vivo* response of peritoneal wall macrophages to laser-induced focal injury suggests a two-step process, in which macrophage processes initially move toward the injury via purinergic signaling, and then require receptor for advanced glycation endproducts (RAGE) for ultimate convergence (Uderhardt et al., 2019). Whether a similar two-stage process in microglia remains to be investigated. There are numerous molecules released from damaged cells that elicit immune responses, collectively known as damage-associated molecular patterns (DAMPs), which include DNA, reactive oxygen species, and proinflammatory proteins (Kigerl et al., 2014). For example, in sterile injury in zebrafish embryos, the presence of a gradient of H_2_O_2_ is required for recruitment of leukocytes to the injury site (Niethammer et al., 2009). Whether H_2_O_2_ or other DAMPs regulate P2Y12-independent motility in the context of laser-induced injury remains unknown. P2Y12-independent motility, as well as different modes of microglial motility more generally, warrants further investigation.

### Interaction between microglial P2Y12 and adrenergic signaling

Several recent studies both *in vivo* and *ex vivo* have shown that the G_i_-coupled P2Y12 receptor and Gs-coupled β2-AR have opposing roles regulating microglial morphology and dynamics, suggesting cAMP has a key role in regulating microglia function. *In vivo* or in acute brain slices, extracellular ATP causes extension of microglial processes in the form of bulbous end tips, via P2Y12 (Davalos et al., 2005; Haynes et al., 2006; Gyoneva and Traynelis, 2013; Dissing-Olesen et al., 2014). In contrast, activation of β2-AR leads to microglial process retraction and reduced tissue surveillance (Gyoneva and Traynelis, 2013; Liu et al., 2019; Stowell et al., 2019). With elevated cAMP, as would be expected with β2-AR stimulation, larger microglial processes retract while highly motile thin filopodial processes become more abundant (Bernier et al., 2019).

The activation of these antagonistic pathways likely controls microglial roles in the remodeling of synapses during activity-dependent plasticity. This is supported by the idea that neuronal activity controls the release of their ligands, ATP and norepinephrine (NE). In acute slices, activation of NMDA-type glutamate receptors is sufficient to elicit P2Y12-dependent microglial process extension (Dissing-Olesen et al., 2014; Eyo et al., 2014). *In vivo*, chemogenetic excitation of neurons induces microglial process recruitment to neuronal cell bodies (Cserép et al., 2020). Furthermore, in line with ATP being a ubiquitous signaling molecule that can act very locally, NMDA receptor activation of a single neuron is sufficient to induce process extension (Dissing-Olesen et al., 2014). Ocular dominance plasticity requires both NMDA receptors (Cooke and Bear, 2014) and microglial P2Y12 (Sipe et al., 2016), suggesting this form of experience-dependent plasticity may rely on NMDA-mediated ATP-dependent recruitment of microglial processes to specific neurons or synapses. In contrast, β2-ARs are activated by NE, a neuromodulator predominantly synthesized by neurons located in the locus coeruleus (LC) with long range projections throughout the brain (Schwarz and Luo, 2015). Increased activity of the LC (representative of an alert brain state), leads to release of NE, which acts on microglial β2-ARs to induce process retraction and impaired surveillance (Liu et al., 2019; Stowell et al., 2019). Activation of microglial β2-ARs blocks ocular dominance plasticity suggesting that noradrenergic regulation of microglia regulates synaptic plasticity (Stowell et al., 2019). Thus, β2-AR signaling may reflect the regulation of microglia by large-scale state-dependent changes in brain activity while P2Y12 signaling allows microglia to respond to stimuli on the subcellular level, both of which influence microglial surveillance and experience-dependent synaptic plasticity. While the idea that β2-AR signaling directly counteracts P2Y12 in this context has not been tested, PI3Kγ is differentially regulated by Gs (through PKA) and G_i_ signaling (Perino et al., 2011; Vanhaesebroeck et al., 2012), and thus could mediate some of the antagonistic effects of P2Y12 and β2-AR signaling in microglia. However, we found that loss of PI3Kγ does not affect microglial surveillance (Fig. 3) or ocular dominance plasticity (Fig. 7), and has only subtle effects on ATP-mediated directed motility (Figs. 4-6). Thus, it is likely not a point of convergence of P2Y12 and β2-AR signaling in microglia *in vivo*.

The intracellular effectors of the multifaceted microglial P2Y12 receptor remain largely unknown. While PI3Kγ has a subtle role in ATP-mediated directed motility, we found that it was not a major mediator of P2Y12 signaling. Future studies will have to determine how P2Y12 activation is translated into microglial movement to better understand its complex role in the CNS.

## Synthesis

Reviewing Editor: Lindsay De Biase, University of California, Los Angeles Decisions are customarily a result of the Reviewing Editor and the peer reviewers coming together and discussing their recommendations until a consensus is reached. When revisions are invited, a fact-based synthesis statement explaining their decision and outlining what is needed to prepare a revision will be listed below. The following reviewer(s) agreed to reveal their identity: NONE.

Both reviewers agree that this is a well-executed study that investigates key mechanistic underpinnings of microglial process motility. Specifically, they investigate the role of PI3Kγ in the signal transduction pathway downstream of P2Y12 after activation by ATP/ADP in microglia. Both reviewers also highlight the use of brain slice and in vivo imaging approaches to investigate this question as a major strength of the study. However, both reviewers agree that the results are not completely negative, but, rather, suggest that PI3Kγ may modulate key aspects of microglial motility in subtle but significant ways. Accordingly, the title, significance statement, and text of the results should be modified to more accurately represent this aspect of the study findings.

Due to the surprising finding that PI3Kγ does not dramatically impact microglial process motility and convergence, it is suggested that using pharmacological approaches to investigate at least one additional potential P2RY12-dependent downstream signaling pathway would greatly enhance the study - particularly if it can identify synergistic interaction between PI3Kγ and these signaling pathways. Nonetheless, such experiments would not be considered an absolute necessity for acceptance of the study.

Reviewer 1:

Understanding the signaling pathway triggered by ATP/ADP activation of P2Y12 in microglia and how this affects process motility, phagocytosis and other microglial outputs would represent a major breakthrough in the field. The mechanism of this process is not well understood, but we now know it is critical for extremely important processes like synaptic pruning, clearance of debris in disease, etc... In this manuscript, the authors describe that the function of PI3Kγ is not required for microglial process motility, including convergence of processes towards a source of ATP and towards a focal laser lesion. It is also not required for baseline process motility, the maintenance of the microglial ramified morphology or for ocular dominance plasticity, a process previously shown to require microglial input. The experiments are well done and well presented, and involve studying microglia either in brain slices or in vivo, which is critical since microglia are highly affected when grown in culture. The analysis tools used to quantify different aspects of microglial physiology are of high quality, especially their analysis of directional velocity and convergence.

Studies showing negative results are very worthwhile, especially in this case where the initial hypothesis (that PI3Kγ would be involved in signaling downstream of P2Y12 activation) is clear and well thought out. However, given that the results actually show a small but significant effect of PI3Kγ absence/inhibition in some experiments, it makes the take-home message confusing on the role of PI3Kγ. Furthermore, it hints at the fact that PI3Kγ is actually involved in the overall responses induced by P2Y12 activation, and therefore warrants further investigation. Since multiple other downstream pathways could play a role in mediating P2Y12 activation, it would make the manuscript much more impactful if the authors could explore at least one of these additional pathways (some are already mentioned in the discussion). This would reveal the relative importance of PI3Kγ vs other potential pathways and may identify synergistic interactions between PI3K (or PI3Kγ) and other major signaling systems induced by ATP/P2Y12 activation. Given that the clear and quantifiable assays the authors have set up, it seem like a perfect opportunity for such investigations.

Major points.

One potential second messenger pathway that was not tested or discussed is the fact that ATP was shown (by the Garaschuk and Tvrdik groups, among others) to elicit strong intracellular calcium increases in microglia. This has been shown in paradigms (laser lesion, ATP puff in slices, etc...) very similar to the ones used here. This calcium signal is likely P2Y12-dependent. Although the exact mechanism remains unknown, it is likely to involve phospholipase C (PLC)-dependent formation of IP3, which requires PIP2 as a substrate. Since the balance between PIP2 and PIP3 is mostly driven by PI3K, it would be interesting to test how calcium responses to ATP (lesion or puff) are affected by PI3K inhibition (wortmannin used in figure 1), or specifically PI3Kγ inhibition or KO.

Arguably the second most ubiquitous second messenger system is intracellular cAMP. Although briefly mentioned in the discussion, the authors should include in their interpretation the fact that P2Y12 is primarily a Gi-coupled GPCR, in that it induces inhibition of adenylate cyclase and cAMP production. This pathway is likely involved in the overall process triggered by P2Y12 activation. Blocking cAMP production may help uncover the role played by PI3Kγ, especially given its potential role in the spatial restriction of cAMP signaling by complexing PKA and PDE3 as discussed on lines 674-679. Multiple pharmacological blockers exist for different targets in the cAMP-PKA pathway and could be combined with PI3K or PI3Kγ inhibition/KO in the various paradigms used by the authors in this manuscript. It may also be worthwhile to discuss the possibility that EPAC is part of the effector pathway downstream of P2Y12 activation, although very few pharmacological agents can be used to investigate that directly.

P2Y12 activation by ATP or lesions was also shown to evoke a potassium current in microglia (by Long-Jun Wu’s group), partially via Gβγ. Also, based on recent work by Attwell’s group, potassium currents are also critical for the ramified morphology of microglia. Blocking the K+ current component of the P2Y12 response could help uncover a role by, or synergistic interaction with PI3Kγ.

Minor points.

Lines 81 and 418. The authors cite Zhang et al. to show that microglia are specifically enriched for P13Kγ mRNA transcripts. Single cell datasets can be highly variable, due to many reasons including different cell sorting strategies. They should also cite other publicly available single cell RNAseq databases like the ones from the Betsholtz group (http://betsholtzlab.org/VascularSingleCells/database.html) or the Linnarsson group (http://mousebrain.org/genesearch.html). These two resources also show highly enriched PI3Kγ signal in microglia, hence that would bolster the author’s statements.

While this would require substantial work and would likely show results similar to those included in this manuscript, it would be interesting to use the CX3CR1-Cre; PI3Kγ fl/fl mice for experiments measuring process convergence towards an ATP source or a focal laser lesion. I assume the earlier work (chronologically) in the project was done with the pan KO and the more recent work was done with the microglia-specific KO?

If the role of PI3Kγ is kinase-independent, as an AKAP complexing PKA and PDE3 as suggested in the discussion, it would be worthwhile to verify whether disrupting AKAP pharmacologically would have the same effect. Several pharmacological agents can disrupt AKAP complexing. Also PI3Kγ can interact with, and regulate specifically the PDE3B subtype of PDE3, which was recently shown (MacVicar group) to be the predominant PDE in controlling cAMP signals in microglia. This should be discussed. On the same topic, if PI3Kγ actions are kinase-independent, would the specific inhibitor used (IPI-549) block the AKAP function of PI3Kγ? Or does it only block the kinase activity?

Line 488 and other instances. Although the term chemotaxis, technically speaking, can refer to the movement of only part of a cell (like a process), it is more commonly used to refer to whole cell movement. Especially in the case of microglia where it is often used in cell culture to refer to whole cell motility towards a source of ATP or other chemoattractant, as in multiple papers cited in this manuscript. The authors use an arguably better and more physiologically representative model by using brain slices or in vivo imaging, they should refer to process movement or convergence as such, to differentiate their experiments from those in cell culture and to prevent misunderstanding of process convergence vs whole cell motility.

The visual abstract could be more informative if other potential pathways were shown along with their potential interactions with PI3Kγ.

Figures 2A, 3B,C and C,D. Groups are labelled PI3Kg instead of PI3Kγ.

Reviewer 2:

This is an interesting contribution by Ania Majewska’s group attempting to investigate the role for PI3kγ in microglial morphology, density, basal and directed process dynamics and ocular dominance plasticity. The study is well and clearly written and the findings are of interest. However, the study would be better improved by the following revisions:

1. The Title: Although chemotaxis and “synaptic plasticity” are an important thrust of the study, they are not the only focus of the study. Thus, the current title is a bit insufficient. This reviewer suggests a more representative title such as “Phosphoinositide 3-kinase γ is not a predominant regulator for static and dynamic microglial responses or experience dependent ocular dominance plasticity”. We suggest this title because the authors show “subtle” roles for PI3kγ, though these don’t seem to be primary. Also, the authors look beyond chemotaxis and include density and morphological analyses in the study. Finally, while ODP is studied, structural plasticity is not investigated and a role for such cannot be ruled out. Moreover, other forms of plasticity were not tested. Therefore, the claim should be more modest towards ODP in specific rather than “synaptic plasticity” in general.

2. Central conclusion: the authors make a quite modest conservative conclusion as stated in the Abstract, Significance Statement, Introduction and throughout the main text that PI3kγ does not play a role in the phenomena examined. We think this does not accurately represent the data since as the authors note, there are subtle (though not major) effects with PI3kγ inhibition/deletion. Thus, the authors should modify these claims (as suggested for the Title in point 1 above) to more accurately reflect the conclusion. To say in one sentence that “...PI3kγ is not required...” and then say in the next sentence that “...the absence or inhibition of PI3kγ had an intermediate effect...” seems a little contradictory.

3. In the Methods, there are several places where the word “REDACTED” is present and should be revised appropriately e.g. on line 109 of page 6 and line 231 on page 10.

4. On lines 463-467, the authors describe how the current study shows a “trend” towards a less ramified process network P2Y12 KO microglia are when compared to wildtype and link this finding to their previous publication in Sipe et al., 2016. However, that earlier study showed a significantly reduced ramified process network in these mice. Can the authors explain the seeming discrepancy in these results?

5. In lines 472-473 the mention of P2y12 roles in microglial dynamics should also cite the original work showing this form Haynes et. al., 2006.

6. In lines 482-483, a comparison between P2Y12 KO (rather than WT) and PI3kγ KO is not clear. This comparison leads to a wrong conclusion that there is a “slight decrease” in surveillance in PI3kγ KOs. This should be rectified.

7. For the studies in Fig. 4, it is not clear whether these drugs were used to determine their effect on microglia in basal conditions i.e. prior to ATP treatment or laser-induced injury.

8. It would be helpful to confirm that IPI-549 at the concentrations used and in the author’s system is indeed sufficiently blocking PI3kγ function. Could the authors look at a different PI3kγ-dependent activity and show that it is blocked by this drug treatment. Else, it could be possible that a higher concentration of the drug may elicit strong effects.

9. The observation in Fig. 6 and Movie 8 that P2Y12 KO microglia show robust directed chemotaxis at a distant farther away from the core injury site is very intriguing and the magnitude of this responses as not been reported before (to this Reviewer’s known and not even by Haynes et. al., 2006 who first reported a role for P2Y12). The authors could speculate more on the meaning of this observation and potential P2Y12-independent mechanisms. Although the authors speculate that these may be purinergic dependent (lines 742 - 743), the lack of such a response in ATP-pipette experiments suggest otherwise and that the mechanisms must be purine-independent. The authors should rectify this and perhaps provide alternative suggestions.
