# Supplementary material for: Phosphoinositide-3-Kinase γ Is Not a Predominant Regulator of ATP-Dependent Directed Microglial Process Motility or Experience-Dependent Ocular Dominance Plasticity
Source: eNeuro. 2020 Dec 21;7(6):ENEURO.0311-20.2020. doi: 10.1523/ENEURO.0311-20.2020 (PMC7769883; doi:10.1523/ENEURO.0311-20.2020)
Supplement: Extended Data 1 — Code accessibility: code used for image analysis. Description of each file is included in the attached document. Download Extended Data 1, ZIP file. [file enu-eN-NRS-0311-20-s20.zip › eneuro_code/eneuro_codedescription.docx]

Matlab and ImageJ macro code to conduct microglial image analysis:

Spacing index (figure 2C):

- Nearestneighborscript.m: Matlab script to calculate nearest neighbor lengths given a set of coordinates.

Sholl analysis for microglia morphology (figure 2E-G):

- Three step process in ImageJ/FIJI and Matlab.
- Sholl_pre_multichannel_0612.ijm: preprocessing confocal z-stacks for Sholl analysis to perform maximum z-projection, crop individual microglia, and apply a binary threshold.
  - Input folder: contains z-stacks of confocal images of microglia
  - Zmax output: folder where maximum z-projections and microglia regions of interest are outlined.
  - Cropped Microglia output: folder where individual thresholded microglia images are shared.
- Sholl_post_0612.ijm: Runs “Sholl Analysis” (FIJI plugin) on each thresholded microglia and saves the intersection values in individual .csv files.
  - Input folder: cropped microglia: see above.
  - Output folder: spreadsheets.
- Sholl_extractdata.m: extracts intersection data from .csv files.
  - Before running, sort the .csv files into folders by animal. The input for this function is the parent directory containing these folders.

Microglia motility analysis (Figure 3):

- Motility_analysis_batch.m: Runs motility and surveillance analysis on xyt movies.
  - Input: folder containing time-series movies of microglial motility.

Directed microglial velocity and convergence analysis (Figures 4-6):

- Series of scripts/functions used to analyze microglial response to focal ATP release or focal ablation. All other functions are called by the ‘Goatland_script_1030’ script.
- Goatland_script_1030: main script that is run by the user.
  - Input: folder containing xyt movies (.tif).
  - Output: will save the output from each step in the analysis to a new folder within this input folder, including a final ‘goat_results’.
  - Calls, in order:
- Goatland_preprocessing_1030: allows user to manually threshold image and select regions of interest (outline focal injury location or select tip of pipet tip and outline pipet).
- Goatland_1030: function that calculates the optic flow vectors from the xyt images.
- Goatland_analysis_1030: applies appropriate masks to optic flow vectors, calculates directional velocity, and calculates averages for image.
- Goatland_postscript_1030: collects values (directional velocity) from previous function for each image and collimates into one matrix.

Directed velocity as a function of distance from center (Figure 6-1):

- Time_distance_plotting: Modification of previous scripts that outputs the directional velocity as a function of distance from the center region.
- Goatland_script_vectordistance.m: similar master script. Input is folder containing xyt movies.
  - Calls Goatland_preprocessing_1030 and Goatland_1030 from above
- Goatland_analysis_vectordistance.m: calculates directional velocity as a function of distance and time for each sample.
- Goatland_postscript_vectordistance.m: collects results from each sample and puts in one single results file.
- Vector_distance_plotting.m: generates heat map plot from results file.
