# Supplementary figures and images for: Phosphoinositide-3-Kinase γ Is Not a Predominant Regulator of ATP-Dependent Directed Microglial Process Motility or Experience-Dependent Ocular Dominance Plasticity
Source: eNeuro. 2020 Dec 21;7(6):ENEURO.0311-20.2020. doi: 10.1523/ENEURO.0311-20.2020 (PMC7769883; doi:10.1523/ENEURO.0311-20.2020)

Supplement: Extended Data 1 — Code accessibility: code used for image analysis. Description of each file is included in the attached document. Download Extended Data 1, ZIP file. [file enu-eN-NRS-0311-20-s20.zip › eneuro_code/helper_functions/TIFFStack-master/private/TS_UnitTestImage.tif]
